# Characteristics of cross-hybridization and cross-alignment of expression in pseudo-xenograft samples by RNA-Seq and microarrays

**DOI:** 10.1186/2043-9113-3-8

**Published:** 2013-04-18

**Authors:** Camilo Valdes, Pearl Seo, Nicholas Tsinoremas, Jennifer Clarke

**Affiliations:** 1 Center for Computational Science, University of Miami, Miami, FL, USA; 2 Department of Medicine, University of Miami, Miami, FL, USA; 3 Division of Biostatistics, Department of Epidemiology and Public Health, University of Miami, Miami, FL, USA; 4 Department of Computer Science, University of Miami, Miami, FL, USA

**Keywords:** Microarray, RNA-Seq, Cross-hybridization, Cross-alignment, Tumor microenvironment, Xenograft, Pathway analysis

## Abstract

**Background:**

Exploring stromal changes associated with tumor growth and development is a growing area of oncologic research. In order to study molecular changes in the stroma it is recommended to separate tumor tissue from stromal tissue. This is relevant to xenograft models where tumors can be small and difficult to separate from host tissue. We introduce a novel definition of cross-alignment/cross-hybridization to compare qualitatively the ability of high-throughput mRNA sequencing, RNA-Seq, and microarrays to detect tumor and stromal expression from mixed ‘pseudo-xenograft’ samples vis-à-vis genes and pathways in cross-alignment (RNA-Seq) and cross-hybridization (microarrays). Samples consisted of normal mouse lung and human breast cancer cells; these were combined in fixed proportions to create a titration series of 25% steps. Our definition identifies genes in a given species (human or mouse) with undetectable expression in same-species RNA but detectable expression in cross-species RNA. We demonstrate the comparative value of this method and discuss its potential contribution in cancer research.

**Results:**

Our method can identify genes from either species that demonstrate cross-hybridization and/or cross-alignment properties. Surprisingly, the set of genes identified using a simpler and more common approach (using a ‘pure’ cross-species sample and calling all detected genes as ‘crossers’) is not a superset of the genes identified using our technique. The observed levels of cross-hybridization are relatively low: 5.3% of human genes detected in mouse, and 3.5% of mouse genes detected in human. Observed levels of cross-alignment are practically comparable to the levels of cross-hybridization: 6.5% of human genes detected in mouse, and 2.3% of mouse genes detected in human. We also observed a relatively high percentage of orthologs: 40.3% of cross-hybridizing genes, and 32.2% of cross-aligning genes.

Normalizing the gene catalog to use Consensus Coding Sequence (CCDS) IDs (Genome Res 19:1316–1323, 2009), our results show that the observed levels of cross-hybridization are low: 2.7% of human CCDS IDs are detected in mouse, and 2.4% of mouse CCDS IDs are detected in human. Levels of cross-alignment using the RNA-Seq data are comparable for the mouse, 2.2% of mouse CCDS IDs detected in human, and 9.9% of human CCDS IDs detected in mouse. However, the lists of cross-aligning/cross-hybridizing genes contain many that are of specific interest to oncologic researchers.

**Conclusions:**

The conservative definition that we propose identifies genes in mouse whose expression can be attributed to human RNA, and vice versa, as well as revealing genes with cross-alignment/cross-hybridization properties which could not be identified using a simpler but more established approach. The overall percentage of genes affected by cross-hybridization/cross-alignment is small, but includes genes that are of interest to oncologic researchers. Which platform to use with mixed xenograft samples, microarrays or RNA-Seq, appears to be primarily a question of cost and whether the detection and measurement of expression of specific genes of interest are likely to be affected by cross-hybridization or cross-alignment.

## Background

It is well understood by oncologists that tumor growth and metastasis depend on changes in the tumor microenvironment or stroma. Stromal changes have been the focus of numerous research publications and have led to insights in both tumor development and promising new avenues for treatment [1-7].

In order to study molecular changes in stroma from tissue samples, it is necessary to separate tumor tissue from stromal tissue. Without this separation we have sample heterogeneity, which is well known to severely limit the conclusions that can be made about the specificity of molecular changes and their biological causes [5,7-11]. This separation can be difficult in contexts where tumors are small or not well differentiated. For example, in mouse tumor xenograft models, human cancer cells are grown in immune-suppressed mice [12-15]. These models are popular in oncologic research for studying mechanisms of tumor growth and metastasis, as well as drug response. In such studies, secondary tumors (at sites of metastasis) are often quite small and difficult to separate surgically from the surrounding stroma. Hence, mouse xenograft samples consist of a mixture of human tumor and mouse stromal tissues, and the task of separating tumor tissue from stromal tissue is a critical one.

Historically, the tumor-stromal separation step has been solved in two different ways: either by laser capture microdissection (LCM; [14,16,17]) of the tissue samples or *in silico* dissection of data from undissected samples [17,18]. LCM is very time consuming, and specialized equipment is required to obtain a sufficient quantity of biological material for profiling. If the sample is in suspension, cell-sorting methods can be used to isolate specific cells, although this requires a suitable biomarker for the cell type(s) of interest. The main drawback of cell-sorting with respect to molecular profiling is that the act of separation itself can alter the expression of molecular markers [10,19,20]. *In silico* dissection can be used successfully to assign expression levels to different tissues; however, these methods may have difficulty with cross-hybridization of human chip probes with homologous mouse genes – and vice versa [21-23].

A potential solution is the use of high-throughput RNA sequencing (RNA-Seq) data to measure expression. RNA-Seq is a relatively new, but very promising method used to detect and measure the abundance of RNAs [2,4,24] using modern DNA sequencing technologies [1,2,4,6,25]. RNA-Seq does not depend on genome annotation for prior probe selection and avoids biases introduced during hybridization of microarrays [8,26]. However, RNA-Seq does depend on alignment of reads to a reference sequence [12,14,24], and current wet-lab RNA-Seq protocols and strategies require lengthy library preparation procedures.

An alternate method for identifying cross-hybridization/cross-alignment would be to profile ‘pure’ human and mouse samples using both mouse and human platforms. Genes detected when examining pure human tissue with a mouse platform, and vice versa, are defined as cross-hybridizers/cross-aligners. This method should identify genes whose expression fluctuates from undetectable to detectable or change their detectable levels when going from a same-species to a cross-species platform. Interestingly, we found that by using a more conservative definition, but a more complex approach – using a titration series to identify genes whose expression shifts from undetectable to detectable – we identified genes that could not be identified using only ‘pure’ species samples.

We report on a qualitative comparison of the abilities of RNA-Seq and microarray data to determine tumor (human) and stromal (mouse) expression from mixed samples in terms of cross-alignment (RNA-Seq) and cross-hybridization (microarrays). We use titration data from experiments designed to create pseudo-xenograft samples, in triplicate, involving total RNA from mouse and human combined in different proportions and analyzed by both microarrays and RNA-Seq.

## Methods

### RNA sources

Samples consisted of normal mouse lung and human breast cancer cells (the human samples consisted entirely on pre-established human cell lines, and did not include any human subjects); these were combined in fixed proportions to create a titration series of 25% steps. The titration data assembles a series of “pseudo-xenograft” samples containing both human and mouse components. MDA-MB-231 human breast carcinoma cell line was obtained from the American Type Culture Collection, and maintained according to the supplier’s instructions. RNA was isolated from three independent cultures of sub-confluent MDA-MB-231 cells in the exponential phase of growth. NOD/SCID gamma mice were obtained from The Jackson Laboratory, and normal lung tissue was harvested from three independent age-matched mice. All animals were maintained in accordance with the guidelines of the Institutional Animal Care and Use Committee at the University of Miami.

RNA was extracted from cultured MDA-MB-231 cells and normal lung tissue using Trizol Reagent (Invitrogen) according to the manufacturer’s instructions. Concentration and yield of RNA samples were determined using a NanoDrop ND-1000 Spectrophotometer (NanoDrop Technologies). RNA integrity was determined by analysis on an Agilent 2100 Bioanalyzer (Agilent Technologies) following the manufacturer’s recommendations. Only samples with a RIN score greater than 7.0 were used for the subsequent molecular analysis.

### Microarrays

Samples were prepared according to the manufacturer’s instructions, and analyzed using HumanWG-6_V3_0_R1 and MouseWG-6_V2_0_R0 Expression BeadChips (illumina, Inc.). Samples consisted of total RNA combined in the following proportions (human/mouse): 100/0, 75/25, 50/50, 25/75, and 0/100. Raw expression data was background corrected, quantile normalized, and log2 transformed using the GeneSpring GX software. Probesets with detection p-values less than 0.05 in two of three independent replicates were considered as “present” and included in further analysis.

### RNA-Seq

mRNA (200 ng) was fragmented at 70 uC for 5 minutes in a fragmentation buffer (Ambion), and converted to first-strand cDNA using Superscript III (Invitrogen); followed by second-strand cDNA synthesis using Escherichia coli DNA pol I (Invitrogen). The double stranded cDNA library was further processed by Illumina Genomic DNA Sample Prep Kit. It involved end repair using T4 DNA polymerase, Klenow DNA polymerase, a T4 Polynucleotide kinase followed by a single “A” base addition using Klenow 39 to 59 exo2 polymerase, and was ligated with Illumina’s adaptor oligo mix using T4 DNA ligase. The adaptor-ligated library was size-selected by separating on a 4% agarose gel and cutting out the library smear at 200-base-pairs (bp) (625 bp). The library was PCR-amplified by Phu polymerase (Stratagene) and purified by Qiaquick PCR Purification Kit (Qiagen). The library was quantified with Quant-iT Picogreen dsDNA Assay Kit (Invitrogen) on a Modulus Single Tube Luminometer (Turner Biosystems) following the manufacturer’s instructions. The resulting library (10 nM) was used to prepare flowcells with approximately 30,000 clusters per lane. Sequencing of all libraries was performed on an Illumina Genome Analyzer IIx yielding 82,447,146 single-end 36-base reads using CASAVA (v.1.3).

The resulting reads were mapped using the TopHat [14,27] spliced-read mapper to the human genome (hg19) and the mouse genome (mm9) obtained from the University of California, Santa Cruz (UCSC) Genome Browser [18,28]. Additionally, the Ensembl [10,13] human and mouse transcriptomes were also supplied to TopHat. Protein-coding genes with at least 5 uniquely aligned reads [16,21] within their respective gene region were considered as being “present” [2,4,19]. We also experimented with aligning – using the BWA aligner [25] – to the transcriptome sequences directly as opposed to the entire genomic reference (Additional file 1: Figures S6 and S7) to gauge any potential advantages or changes in alignment patterns. However we found no perceived benefit as the transcriptome alignment patterns followed the genomic ones, albeit at lower levels.

### CCDS data

Consensus Coding Sequence (CCDS) [29] annotation data was obtained from the CCDS project FTP repository at NCBI. CCDS data was used to normalize the gene catalog in both the microarray and RNA-Seq analyses. For human, 25,504 CCDS IDs were employed; for mouse, 22,131 CCDS IDs were employed in the analysis. The human microarray chip contained 48,811 probes; of these we were able to summarize 21,636 CCDS IDs, of which 16,579 are unique ids. The mouse microarray chip contained 45,282 probes; we summarized them into 20,209 mouse CCDS IDs, of which 13,518 are unique. In review, the human microarray chip contained only 65.0% of current human CCDS IDs; the mouse microarray chip contained only 61.1% of current mouse CCDS IDs.

### Cross-hybridization & cross-alignment

We define a gene which cross-hybridizes (or cross-aligns) from mouse to human (a mouse crosshyb, or mouse cross-aligner) as one which exists in the set defined by ((B ∪ C ∪ D) – A); where “A”, “B”, “C”, and “D” are defined as follows: “A” is the set of all genes detected when the pure mouse RNA sample was hybridized onto mouse chips (or aligned to the mouse genome); “B” is the set of all genes detected when the 25% human & 75% mouse RNA samples were hybridized onto mouse chips (or aligned to the mouse genome); “C” is the set of all genes detected when the 50% human & 50% mouse RNA samples were hybridized onto mouse chips (or aligned to the mouse genome); and finally, “D” is the set of all genes detected when the 75% human & 25% mouse RNA samples were hybridized onto mouse chips (or aligned to the mouse genome). Accordingly, a gene that cross-hybridizes (or cross-aligns) from human to mouse (a human crosshyb, or human cross-aligner) is defined analogously.

## Results

### Intraspecies and interspecies detection

**Figure 1:**
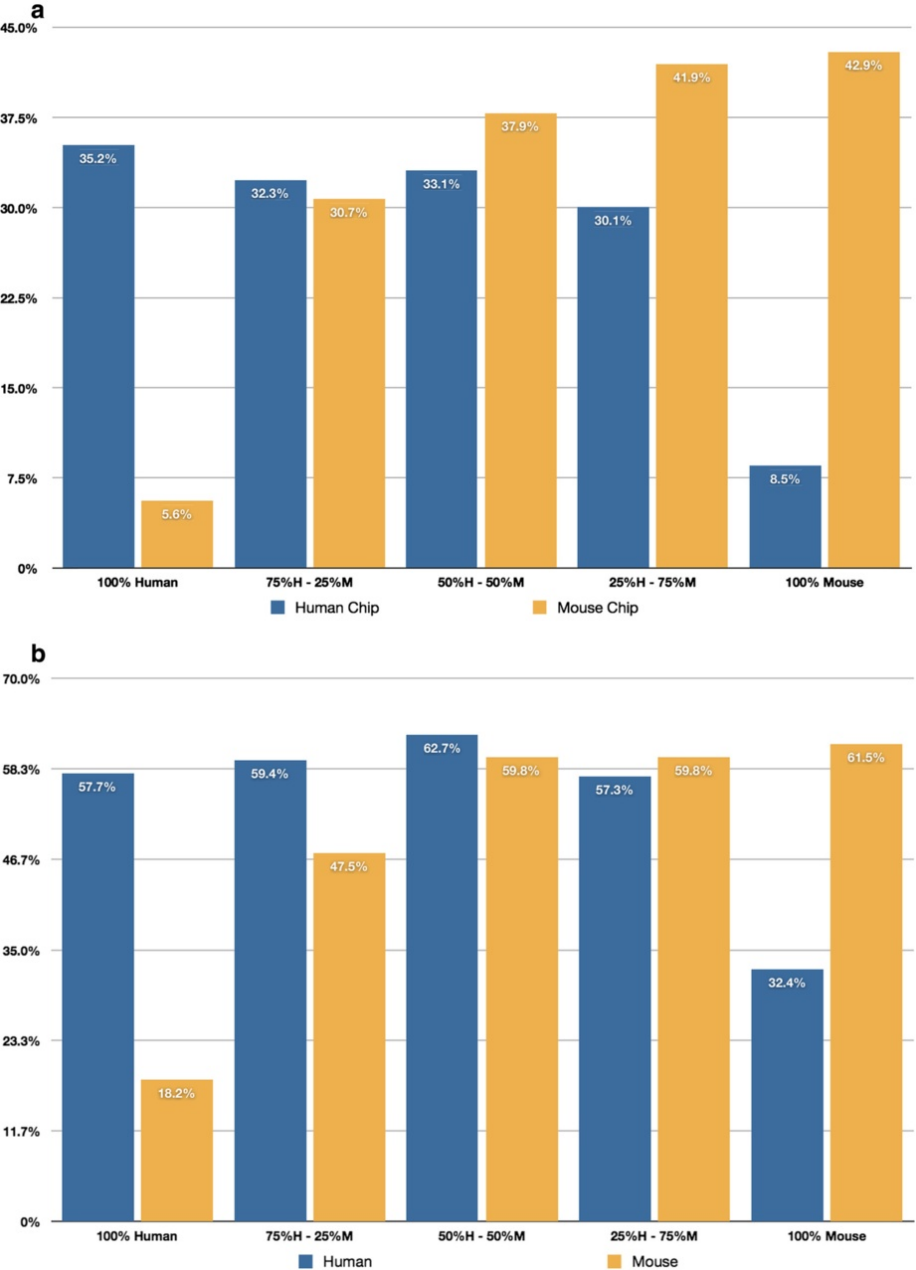
**Human and mouse genes detected by microarrays. a**. Human and mouse genes detected by microarrays. Percentage of genes, on average, within each sample type detected by the microarray chips. Blue bars represent the percentage of human genes that are detected in the human microarray chip; yellow bars represent the percentage of mouse genes detected in the mouse microarray chip. **b**. Human and mouse Ensembl genes detected by RNA-Seq. Percentage of genes detected, on average, within each sample type by RNA-Seq. Blue bars represent the percentage of human genes detected by aligning to the human reference; yellow bars represent the percentage of mouse genes detected by aligning to the mouse reference.

**Figure 2:**
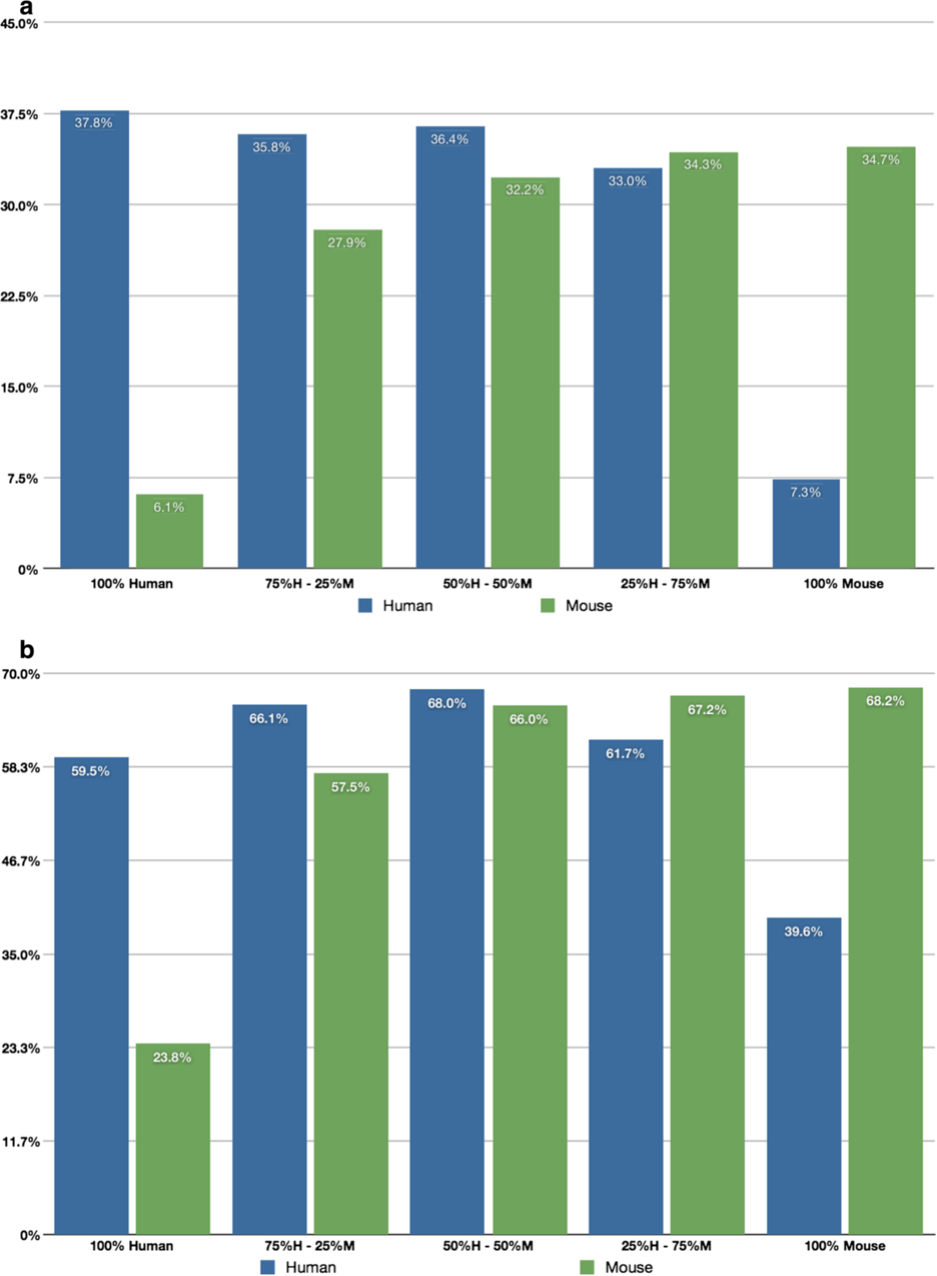
**Human and mouse CCDS Ids detected by microarrays. a**. Human and mouse CCDS IDs detected by microarrays. Percentage of CCDS IDs within each sample type detected by the microarray chips. Blue bars represent the percentage of human CCDS IDs that are detected in the human microarray chip; green bars represent the percentage of mouse CCDS IDs detected in the mouse microarray chip. **b**. Human and mouse CCDS IDs detected by RNA-Seq. Percentage of CCDS IDs detected within each sample type by RNA-Seq. Blue bars represent the percentage of human CCDS IDs detected by aligning to the human reference; green bars represent the percentage of mouse CCDS IDs detected by aligning to the mouse reference.

Figure 1a and b show the percentages of human and mouse genes detected by microarray and by RNA-Seq in each triplicate of samples using a disjoint gene catalog, i.e., the gene list used in the microarray analysis is the entire gene inventory on the chip, and the gene list used in the RNA-Seq analysis is the complete Ensembl protein-coding gene catalog. Figure 2a and b show the comparable results using the CCDS ID catalog for analysis in both technologies. The percent detected is lower overall for the microarray data, i.e., lower within the same species but also lower across species. One can also see that the rate of cross-hybridization of human genes onto the mouse microarray chip is larger relative to the rate of cross-hybridization of mouse genes onto the human microarray chip. A priori we expected that intraspecies detection (human cell line sample hybridized on a human microarray chip, or the human cell line sample RNA-Seq reads mapped onto the human reference; the mouse tissue sample hybridized on a mouse microarray chip, or mouse tissue sample RNA-Seq reads mapped onto the mouse reference) would decrease monotonically as the percentage of same-species material decreased (Additional file 1: Figures S4 and S5); however, this was not the case. In all four cases (mouse tissue on mouse chip, human cell line on human chip, mouse tissue mapped to mouse reference, human cell line mapped to human reference) only the human cell line on the human chip case shows a monotonically decreasing detection rate with decreasing human cell line percentage. For example, the detection of mouse genes by alignment to the mouse reference with the RNA-Seq data actually shows the highest level of detection in samples that are 50%/50% mouse/human cell line. These results may partly be explained by cross-hybridization and/or cross-alignment, i.e., if there is some cross-hybridization and/or cross-alignment then it is possible that heterogeneous samples (mixtures of human and mouse) will have higher detection rates than homogeneous samples (pure mouse and human).

We considered using a much simpler but broader definition of cross-hybridization/cross-alignment. The idea is to define a human cross-hybridizer as a gene detected in a 100% human sample with a mouse microarray, and a mouse cross-hybridizer as a gene detected in a 100% mouse sample with a human microarray. The definitions from cross-alignment would be analogous. We expected that these definitions would lead to lists of genes that would be a superset of the lists we had found, because this simpler definition should identify genes either detected or undetected by same-species methods and detected by cross-species methods, while our definition only identifies genes undetected by same-species methods and detected by cross-species methods. However, we found that neither list was a proper subset of the other; for example, 45.3% (Additional file 1: Table S6) of genes we defined as human cross-aligners by our definition also appeared as a human cross-aligner by the simpler, ‘pure’ sample approach. Additional file 1: Tables S6-S10 contain the full overlap percentages for genes and CCDS IDs detected using both definitions.

### Detection by technology

The CCDS gene catalog was employed so that we may directly compare detection by the two technologies. We used the full set of CCDS IDs obtained from NCBI: 25,504 human CCDS IDs and 22,131 mouse CCDS IDs. It should be noted that the microarray chips only contained 65.0% of the current human CCDS IDs and 61.1% of the mouse CCDS IDs. Additional file 1: Table S2 details the levels of detection by each technology at each sample. RNA-Seq is able to detect more unique CCDS IDs by itself than the microarray chips in the heterogeneous samples: in human, RNA-Seq is able to uniquely detect, on average, about 48.8% of CCDS IDs while the microarray chips are able to uniquely detect only 4.7%; however both technologies detect about 46.5% of the same CCDS IDs. The detected numbers are similar in mouse: RNA-Seq is able to uniquely detect 53.1% of CCDS IDs, while the microarrays are able to uniquely detect 5.4%; both technologies are able to detect 41.5% of the same CCDS IDs (see Additional file 1: Figure S1).

The detection in the Homogeneous samples also follows the same pattern within a species (Venn Diagrams in Additional file 1: Figure S2): for the 100% human sample, RNA-Seq uniquely detects 41.5% of unique human CCDS IDs, microarrays uniquely detect 7.8%, and both detect 50.7% of human CCDS IDs. For the 100% mouse sample, RNA-Seq detects 51.0% of mouse CCDS IDs, microarrays detect 3.8%, and both detect 45.2% of mouse CCDS IDs. However, RNA-Seq detects a substantially greater number of CCDS IDs in the 100% sample of the opposite species: in the 100% mouse sample, RNA-Seq is able to detect 82.7% of human CCDS IDs, while microarrays only detect 7.1% and 10.1% are detected by both. The same is true for the 100% human sample: RNA-Seq is able to detect 77.5% of mouse CCDS IDs, while microarrays are able to detect only 12.2% of mouse CCDS IDs and only 10.2% are detected by both.

**Figure 3:**
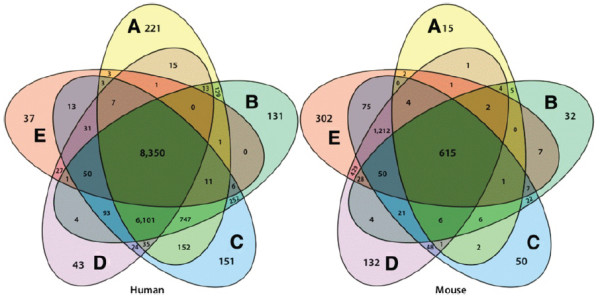
**Detection by both technologies. **Symmetrical Venn-diagrams of CCDS ID’s detected by both RNA-Seq and microarrays in human and mouse. ‘A’ is the 100% human sample, ‘B’ is the 75% human and 25% mouse sample, ‘C’ is the 50% human and 50% mouse sample, ‘D’ is the 25% human and 75% mouse sample, and ‘D’ is the 100% mouse sample. The middle region is the number of CCDS IDs that are detected across all samples.

For the 100% human sample, 16,463 CCDS IDs are detected by both RNA-Seq and microarrays; of these 6,833 are uniquely detected by RNA-Seq, 8,350 are detected by both technologies, and 1,280 are uniquely detected by microarrays. For the 100% mouse sample, 15,696 mouse CCDS IDs are detected by both RNA-Seq and microarrays; of these 8,008 are uniquely detected by RNA-Seq, 7,093 are detected by both technologies, and 595 are uniquely detected by microarrays. The symmetrical Venn Diagrams (n = 5) in Figure 3 illustrates a detailed breakdown of the detection numbers by both technologies in each sample and for each species.

### Levels of cross-hybridization and cross-alignment

**Figure 4:**
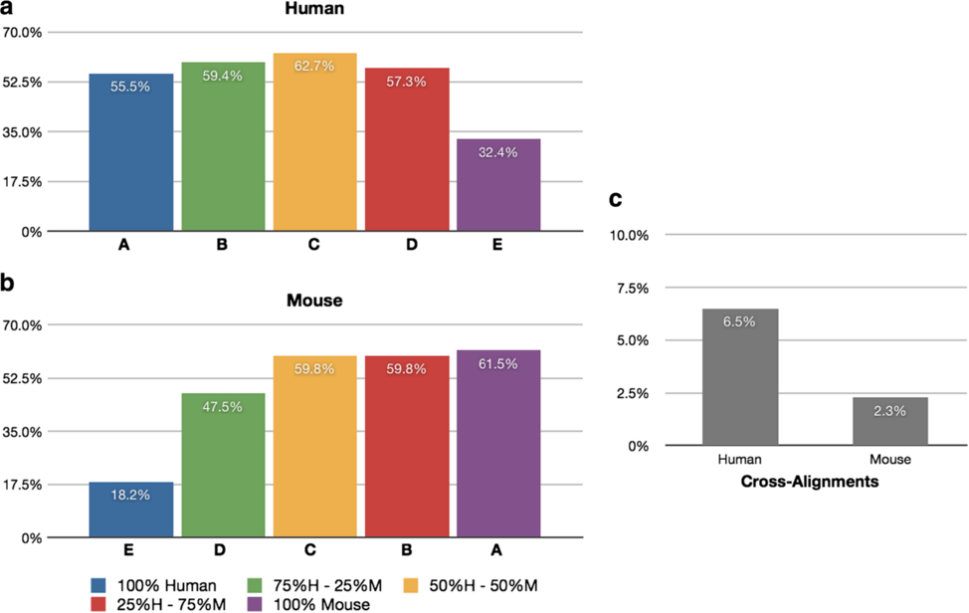
**Gene levels of cross-hybridization. **Cross-hybridizing detected genes from the disjoint gene catalog using the microarray platform. (**a**) Percentage of human genes that are detected in each sample using the human microarray chip. (**b**) Percentage of mouse genes that are detected in each sample using the mouse microarray chip. (**c**) genes that cross-hybridize are identified by subtracting the genes detected in a homogeneous tissue sample (the “A” set) from the union of the mixed tissue samples (B,C, &D).

Figure 4a shows the percentage of human genes from each sample that are detected on the human microarray chip, while Figure 4b shows the analogous information for mouse genes detected in the mouse microarray chip. Figure 4c displays the percentage of detected genes that represent cross-hybridization from human to mouse, and also the percentage of genes that represent cross-hybridization from mouse to human. The determination of cross-hybridization is described in the Methods section. Briefly, genes that cross-hybridize from human to mouse are defined as follows. We consider all genes that are detected on the human chip in 2 of 3 independent replicates of a mixed tissue sample (any combination of mouse and human). We then subtract any genes that are detected on the human chip in 2 of 3 independent replicates of a homogeneous sample (all human). Genes that cross-hybridize from mouse to human are defined similarly. In this way we identify as cross-hybridizing all genes that are detected in mixed human & mouse samples, but not detected in the pure human & mouse samples.

**Figure 5:**
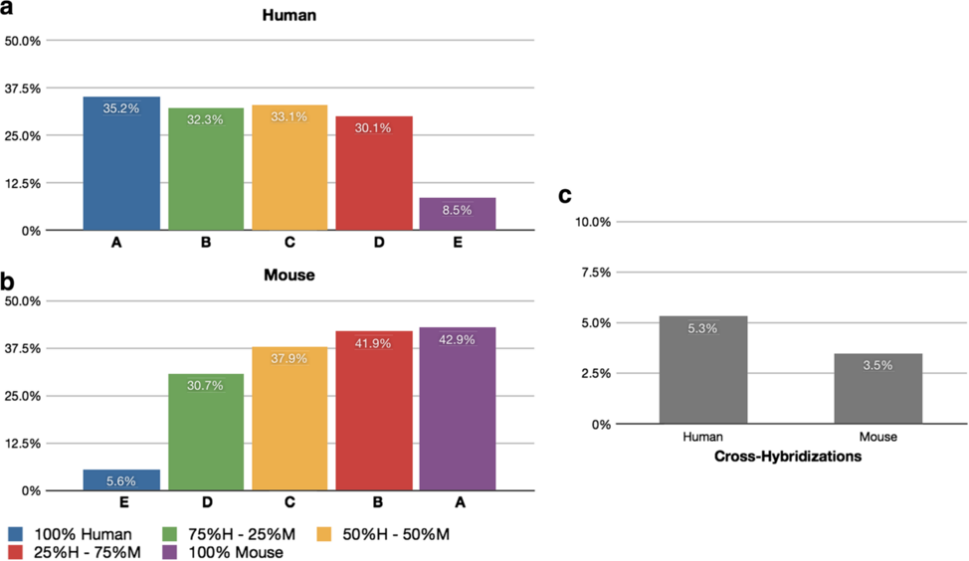
**Gene levels of cross-alignment. **Cross-aligning genes detected from the disjoint gene catalog using RNA-Seq. (**a**) Percentage of human genes that are detected in each sample when aligning to the human reference. (**b**) Percentage of mouse genes that are detected in each sample when aligning to the mouse reference. (**c**) genes that cross-align are identified by subtracting the genes detected in a homogeneous tissue sample (the “A” set) from the union of the mixed tissue samples (B,C, &D).

Figure 5a shows the percentage of human genes that are detected using RNA-Seq in the human reference, while Figure 5b shows information analogous to Figure 5a, but for mouse genes detected in the mouse reference. Figure 5c displays the percentage of detected genes that represent cross-alignment from human to mouse, and also the percentage of cross-aligning genes from mouse to human. Our definition of cross-alignment is also described in the Methods section. In brief, the cross-aligning genes from human to mouse are established as follows. We consider all the genes that are detected in the human reference and transcriptome mappings, in 2 of 3 replicates of a mixed tissue sample. We then subtract any genes that are detected in the human reference and transcriptome mappings in 2 of 3 independent replicates of the homogeneous sample (all human). Cross-alignments from mouse to human are defined similarly.

**Figure 6:**
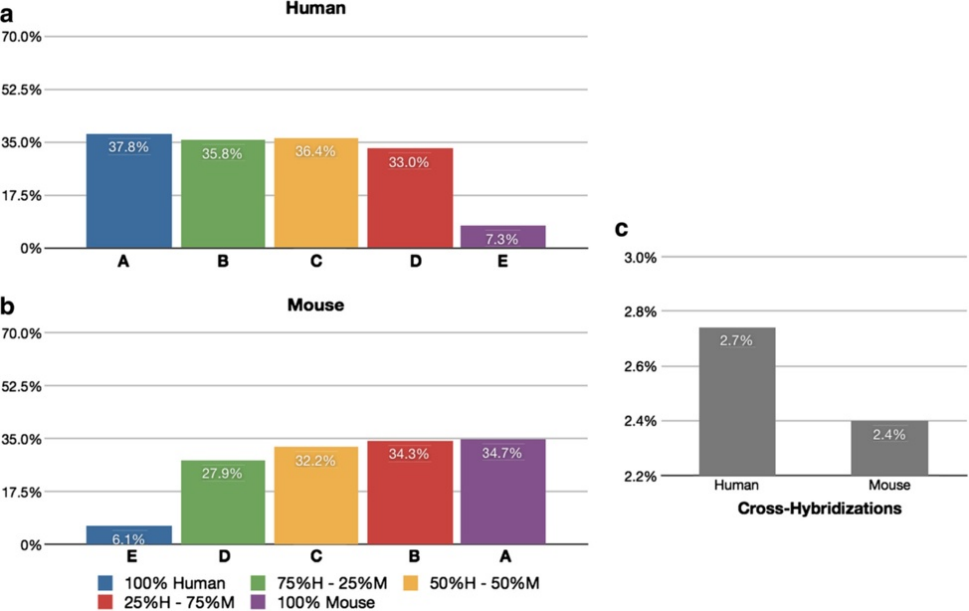
**CCDS levels of cross-hybridization. **Cross-hybridizing CCDS IDs detected using the microarray platform. (**a**) Percentage of human CCDS IDs that are detected in each sample using the human microarray chip. (**b**) Percentage of mouse CCDS IDs that are detected in each sample using the mouse microarray chip. (**c**) CCDS IDs that cross-hybridize are identified by subtracting the CCDS IDs detected in a homogeneous tissue sample (the “A” set) from the union of the mixed tissue samples (B,C, &D).

**Figure 7:**
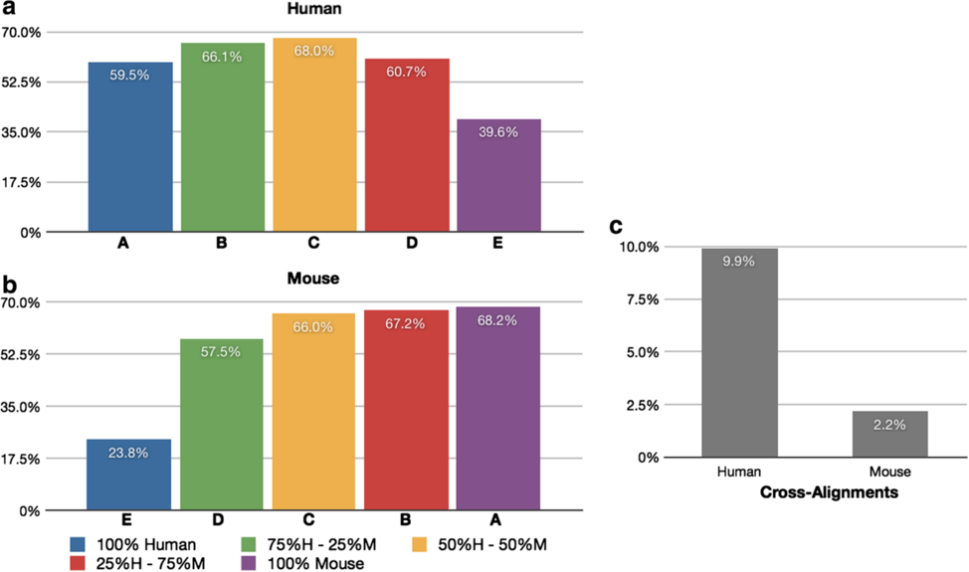
**CCDS levels of cross-alignment. **Cross-aligning CCDS IDs detected using RNA-Seq. (**a**) Percentage of human CCDS IDs that are detected in each sample when aligning to the human reference. (**b**) Percentage of mouse CCDS IDs that are detected in each sample when aligning to the mouse reference. (**c**) CCDS IDs that cross-align are identified by subtracting the CCDS IDs detected in a homogeneous tissue sample (the “A” set) from the union of the mixed tissue samples (B,C, &D).

The observed levels of cross-hybridization in the microarray chips in the case of a disjoint gene catalog are relatively low: 5.3% of human protein coding genes detected in the mouse sample, and 3.5% of mouse protein coding genes detected in the human samples. When we analyze the data using the normalized CCDS ID catalog (Figure 6 and Additional file 1: Figure S3) the levels of cross-hybridization are also low: 2.7% of human CCDS IDs are cross-hybridizing with mouse tissue, and 2.4% of detected mouse CCDS IDs are cross-hybridizing with human. This is consistent with the microarray chip manufacturer’s intent to design gene probes that are species specific, i.e., the chips will detect the gene segment of interest in either mouse or human, but do not detect genes in both species. With RNA-Seq, using a disjoint gene catalog, the observed levels of cross-alignment are practically similar to the levels of cross-hybridization in the microarray chips: 6.5% of human protein coding genes are detected using the mouse reference, and 2.3% of mouse protein coding genes are detected using the human reference. Utilizing the CCDS data (Figure 7), the levels of cross-alignment are very different for the human set: 9.9% of detected human CCDS IDs are cross-aligning with mouse tissue; however, only 2.2% of detected mouse CCDS IDs are cross-aligning with human. This is in contrast to the cross-hybridizing results in which the levels were comparable for both human and mouse set. We posit that the difference is due to the gene model completeness in the mouse transcriptome relative to that of the human transcriptome [26] as well as the higher dynamic range of RNA-Seq [24]. The observed levels of cross-hybridization and cross-alignment are statistically significantly different (test of proportions; p < 0.05), with significantly lower human cross-hybridization than cross-alignment, and significantly higher mouse cross-hybridization than cross-alignment. In practical terms for all cases except human CCDS cross-alignment the percentages seem quite close and the strong statistical significance is likely due to the very large number of genes detected (i.e., very large sample sizes). Lists of cross-aligning and cross-hybridizing genes for human and mouse can be found in Additional files 2 and 3 respectively.

### Orthologs

We examined the number of human-mouse orthologs present in the cross-hybridizing and cross-aligning gene lists. Human-mouse ortholog data was obtained from the Mouse Genome Database (MGD; [27]). The MGD database catalogues 17,673 gene-orthologs between human and mouse. We accumulated all the cross-aligning genes from the disjoint catalog analysis and obtained 1,840 cross-aligning genes. Using these 1,840 cross-aligning genes (human genes detected in the mouse tissue, and mouse genes detected in the human cell line) from the disjoint RNA-Seq results, we identified 593 human-mouse orthologs in the MGD catalog. That is, from the cross-aligning genes, 32.2% were orthologs. For the CCDS data, we identified 16,679 mouse CCDS IDs and 16,902 human CCDS IDs in the MGD database; of these, 16,292 were perfectly paired as human-mouse orthologs. Using the 2,530 cross-aligning human CCDS IDs along with the 481 cross-aligning mouse CCDS IDs, we identified 1,934 human-mouse orthologs: 1,566 CCDS IDs identified from the human cross-aligning set, and 368 identified from the mouse cross-aligning set. In total, 61.9% of the detected human cross-aligning CCDS IDs and 76.5% of the detected mouse cross-aligning CCDS IDs were human-mouse orthologs.

In the microarray analysis, we collected 4,171 cross-hybridizing genes from the disjoint catalog analysis (human genes detected in the mouse tissue, and mouse genes detected in the human cell line), and identified 1,682 orthologs in the MGD catalog. That is, from the cross-hybridizing genes, 40.3% were orthologs. In the CCDS analysis, we collected 699 cross-hybridizing human CCDS IDs and 531 mouse CCDS IDs; from these, we identified 499 orthologs present in the human set, and 419 present in the mouse set. In total, 71.4% of the detected human cross-hybridizing CCDS IDs and 78.9% of the detected mouse cross-hybridizing CCDS IDs orthologs.

Of those orthologs found by the RNA-Seq protocol, 80.6% were detected only in human, 17.3% were detected only in mouse, and 2.1% were detected in both. For microarrays, 53.9% of the orthologs were detected only in human, 45.1% were only detected in mouse, and 1.0% was detected by both. The relatively high percent of orthologs in the detected sets helps explain why, as noted previously, we sometimes observed the highest percentage of genes detected from mixed species samples as opposed to single species samples.

**Figure 8:**
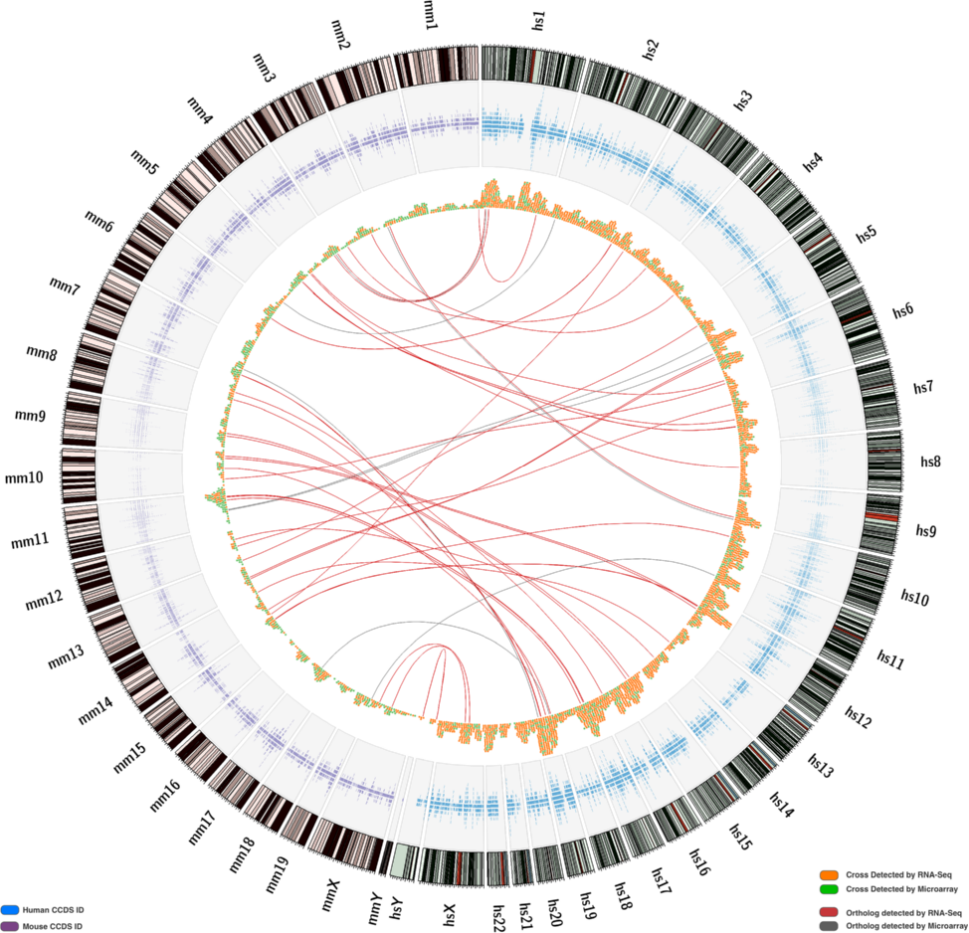
**Orthologs. **Outer bands are human (hs) and mouse (mm) chromosome ideograms. CCDS density across both genomes is depicted in the blue and purple tracks. Orange marks are cross-alignments and cross-hybridizations detected by RNA-Seq; green marks are cross-alignments and cross-hybridizations detected by microarrays. Arcs connect orthologous CCDS IDs that belong to both the cross-alignments and cross-hybridizations sets for each technology – RNA-Seq and microarrays.

Figure 8 contains a Circos [28] plot that displays the results of the normalized CCDS analysis in both the human and mouse genomes. Outer bands represent chromosome ideograms for the human genome (right) and the mouse genome (left). The blue and purple tracks showcase CCDS density for each genome and each chromosome. Levels of cross-alignment and cross-hybridization are presented as orange and green marks in the third inner-most track: orange marks are those CCDS IDs identified by RNA-Seq, and green marks are those CCDS IDs identified by microarrays. The links in the middle of the diagram correspond to the human-mouse orthologs that were identified by each technology: red links represent orthologs detected by RNA-Seq and grey links represent orthologs detected by microarrays.

### Known cancer genes and pathways

**Figure 9:**
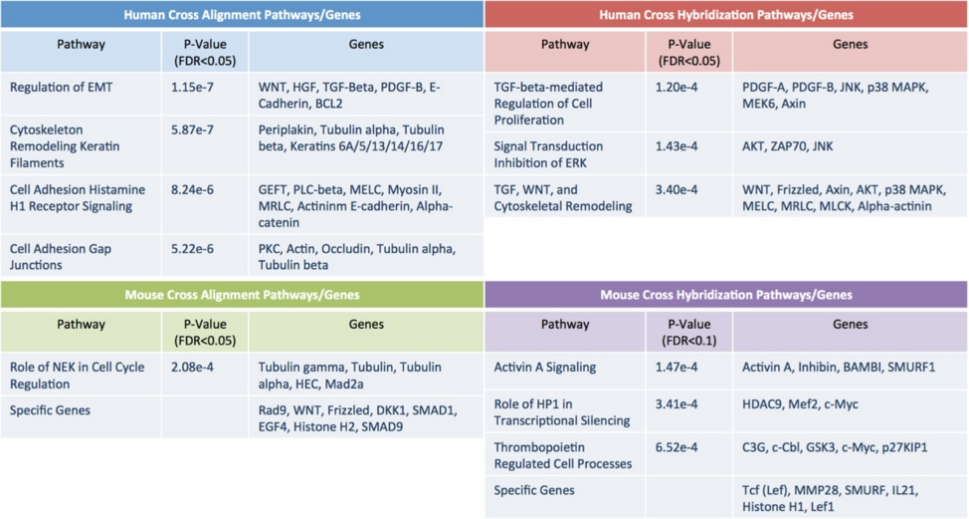
**Enriched cancer gene pathways. **Pathway analysis was performed using MetaCore software from GeneGo Inc. We examined biological pathways over-represented by the genes in each human and mouse cross-hybridizing and cross-aligning CCDS lists. Distinctly different genes and biological pathways appear as cross-hybridizing & cross-aligning depending upon the platform and tissue type.

We examined the cross-hybridizing and cross-aligning gene lists from both the disjoint gene catalog analysis and from the CCDS analysis for genes known to play a role in cancer, or of current interest to researchers in oncology. Cross-hybridizing and cross-aligning genes in our studies include PDGF-B, b-Raf, Beta-catenin, erbB2, NF-kB, MDM2, Claudin, VEGF-R, Notch2, Cyclin B, HSP90 and Ubiquitin. These genes are of particular interest because xenografts are a common laboratory tool in oncology and there is interest in not only expression in tumors, but also in the expression in the stroma. Data from mixed samples will be insufficient for determining whether genes that cross-hybridize and cross-align are being expressed in tumor, stroma, or both. Figure 9 contains several of these genes and the platforms on which they were detected. Full lists of cross-hybridizing and cross-aligning genes are provided in the Additional file 1.

Suppose a sample of mixed tumor and stroma is arrayed on a human platform, with the intent of detecting only tumor-related genes. Genes detected in mouse tissue using human platforms could be perceived as being expressed in tumor, when in fact they are expressed in stroma or are not expressed at all. In our experiments we have found such genes. These include Angiotensin 1-converting enzyme gene (ACE), which has been shown to have possible mitogenic and angiogenic effects in cell line and animal models of breast cancer [13], and heparanase (HPR), which has been proposed as a target for the development of breast cancer directed gene therapy [16]. Genes from the WNT pathway also appear, such as WNT and Axin, that have been implicated as contributing to breast cancer cell proliferation [19]. Conversely, suppose a sample of mixed tumor and stroma is arrayed on a mouse platform with the intent of detecting only stromal genes. Genes detected in the human cell line using mouse platforms could be perceived as being expressed in stroma, when in fact they are expressed in tumor or not expressed at all. An example of such a gene in our experiments is SHC binding protein 1 (SHCBP1), which is regulated by c-Myc and the androgen receptor, and is involved in cell proliferation and growth [22]. Another such gene is Histone H1, which is implicated in breast cancer cell proliferation [30].

For the pathway analysis we used the MetaCore software (GeneGo Inc.) to examine biological pathways over-represented by the genes in each cross-hybridizing and cross-aligning list from both the disjoint catalog analysis and the CCDS analysis. This pathway analysis revealed pathways involved in TGF-beta regulation, cytoskeleton remodeling, regulation of epithelial-mesenchymal transition (EMT), DNA damage, cell adhesion, and FGFR signaling. Select pathways for both analyses are listed in Table 1 (disjoint catalog) and in Table 2 (CCDS). Full pathway results are provided in the Additional file 1.

**Table 1:** Enriched pathways in disjoint catalog analysis

	**Human**		**Mouse**	
	**Cross-alignment**	**Cross hybridization**	**Cross-alignment**	**Cross hybridization**
**1**	Cytoskeleton remodeling: keratin filaments	Protein folding and maturation: POMC processing	Cytoskeleton remodeling: neurofilaments	Neurophysiological process: Dopramine D2 receptor transactivation of PDGFR in CNS
**2**	Signal transduction: cAMP signaling	Normal and pathological: TGF-beta mediated regulation of cell proliferation	DNA damage: ATM/ATR regulation of G2M checkpoint	Transcription role of heterochromatin protein 1 (HP1) family in transcriptional silencing
**3**	Development: regulation of epithelial to mesenchymal transition (EMT)	Cytoskeleton remodeling: TGF, WNT and cytoskeletal remodeling	Cell cycle: role of Nek in cell cycle regulation	Development: hedgehog and PTH signaling pathways in bone and cartilage development
**4**	Cell adhesion: gap junctions	Signal transduction, Erk interaction: inhibition of Erk	Cell cycle: spindle assembly and chromosome separation	Cytoskeleton remodeling: neurofilaments
**5**	Development: transcription regulation of granulocyte development	Development: melanocyte development ang pigmentation	Development: hedgehog signaling	Development: Gastrin in differentiation of the gastric mucosa
**6**	Cytoskeleton remodelling: regulation of actin cytoskeleton by Rho GTPases	Translation: non-genomic (rapid) action of androgen receptor	Mechanisms of CFTR activation by S-nitrosoglutathione (normal and CF)	Blood coagulation: Blood coagulation
**7**	Cell adhesion: Histamine H1 receptor signlaing in the interruption to cell barrier integrity	G-protein signaling : Rap1A regulation pathway	Development: FGFR signaling pathway	Development thrombopotein-regulated cell processes
**8**	Development: beta-adrenergic receptors signaling via cAMP	Development: GDNF family signaling	Muscle contraction: regulation of eNOS activity in endothelial cells	Neurophysiologiacal process: ACM regulation of nerve impulse
**9**	Cytoskeleton remodeling: reverse signaling by Ephrin B	DNA damage: role of SUMO in p53 regulation	GFTR folding and maturation (normal and CF)	Development: WNT5 signaling
**10**	Regulation of lipid metabolism: regualtion of lipid metabolism by niacin and isoprenaline	Muscle contaction: relaxin signaling pathway	DNA damage: role of SUMO in p53 regulation	Transport: Alpha-2 energenic receptor regulation of Ion channels

Enriched GeneGo pathway maps for human and mouse. Gene lists for pathway analsysis were obtained from the disjoint gene catalog analysis for both cross-hybridization (microarrays) and cross-alignment (RNA-Seq).

**Table 2:** Enriched pathways in CCDS analysis

	**Human**		**Mouse**	
	**Cross-alignment**	**Cross hybridization**	**Cross-alignment**	**Cross hybridization**
**1**	Development: regulation of epithelial to mesenchymal (EMT)	Protein folding and maturation: POMC processing	Cell cycle: role of Nek in cell cycle regulation	Signal transduction: activin A signaling regulation
**2**	Cytoskeleton remodeling: Keratin filaments	Normal and pathological TGF-beta mediated regulation of cell proliferation	Cell cycle: the methaphase checkpoint	Transcription role of heterochromatin protein 1 (HP1) family in trancriptional silencing
**3**	Cardia hypertrophy NF-AT signaling in cardia hypertrophy	Signal transduction, Erk interactions: inhibition of Erk	Cytoskeleton remodeling: neurofilaments	Cytoskeleton remodeling: neurofilaments
**4**	Cell adhesion: gap junctions	Development: melaconyte development and pigmentation	ATP metabolism	Dvelopment: thrombopoietin regulated cell process
**5**	Cell adhesion: hestamine H1 receptor signaling in the interruption of cell barrier integrity	Cytoskeleton remodeling: TGF, WNT and cytoskeletal remodeling	Cell cycle: spindle assembly and chromosome separation	Neurophysiological process nNOS signaling in neronal synapases
**6**	Signal transduction: cAMP signaling	Develeopment A2A receptor signaling	dCTP/dUTP metabolism	Development: role of HDAC and calcium/calmodulin dependent kinase (CaMK) in control of skeletal of myogenesis
**7**	Immune response: MIF-neuroendocrine macrophage connector	Development transcription regulation of granulocyte development	Apoptosis and survival: DNA damage induced apoptosis	Protein folding: membrane trafficking and signal transduction of G-alpha heterotrimeic G-protein
**8**	Development transcription: regulation of grabulocyte development	Cytoskeleton remodeling Alpha-1A andrenergic recepto- dependent inhibition of PI3K	Transcription: androgen receptor nuclear signaling	Development: role of activin A in cell differentiation and proliferation
**9**	Cell adhesion: edothelial cell contacts by junctional mechanisms	Muscle contraction: relaxin signaling pathway	Neurophysiological process: ACM regulation of nerve impulse	Development: WNT5A signaling
**10**	Atherosclerosis: role of ZNF202 in regulation of expression of genes involved in Atherosclerosis	Immune response: lectin induced complement pathway	Development WNT signaling pathway	Development: PIP3 signaling in cardiac myocytes

Enriched GeneGo pathway maps for human and mouse. Gene lists for pathway analysis were obtained from the CCDS ID analysis for cross-hybridization (microarrays) and cross-alignment (RNA-Seq).

**Figure 10:**
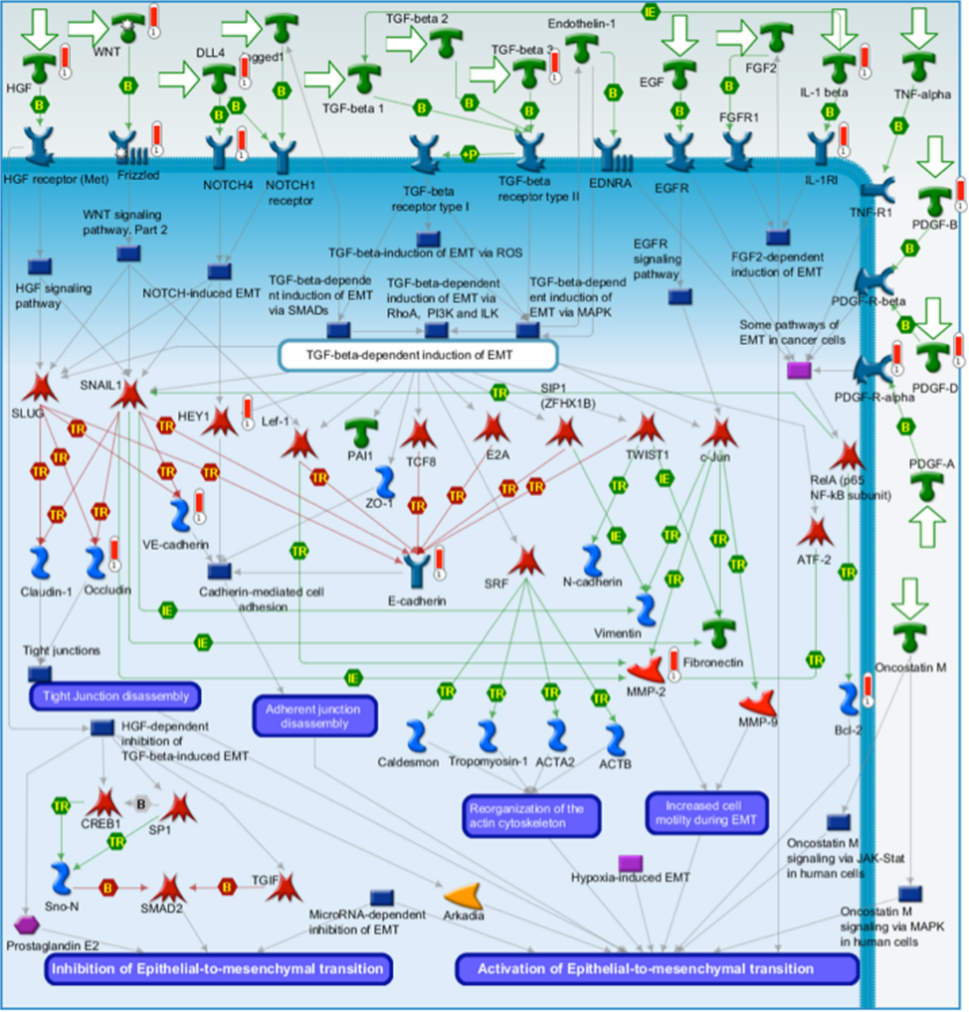
**EMT Pathway. **The top scored GeneGO pathway map (lowest p-value) for the human cross-aligning CCDS set is a development pathway: regulation of epithelial to mesenchymal transition (EMT). Upward thermometers with a red color are up-regulated genes, and downward blue thermometers indicated down-regulated genes.

Distinctly different genes and biological pathways appear as cross-hybridizing/cross-aligning depending upon the platform and tissue type. The cross-hybridizing human genes represent TGF-beta signaling, androgen regulation, and DNA damage, while cross-aligning human genes represent EMT, keratin filaments, and gap junctions. Similarly, cross-hybridizing mouse genes represent HP1 transcription, thrombopoetin processes, and WNT signaling, while cross-aligning mouse genes represent DNA damage, hedgehog signaling, and FGFR signaling. Figure 10 shows the top scored pathway for the cross-aligning human CCDS analysis: regulation of epithelial-mesenchymal transition (EMT) [31,32]. Regulation of EMT is of great interest to cancer researchers, particularly those interested in metastasis, as EMT is a recognized mechanism for initiating metastasis of epithelial cancer cells [33]. The EMT process may facilitate the generation of cancer cells with the mesenchymal traits needed for dissemination as well as the self-renewal properties needed for initiation of secondary tumors. There is accumulating evidence that EMT and mesenchymal-related gene expression are associated with aggressive breast cancer subtypes and poor clinical outcome [34]. Many of the genes from our lists that appear in this and other pathways, have been implicated in tumor-stromal interactions and/or are related to stromal responses to invasion. These include PGDF [35], MMP2 [36], Tubulins [37], and the WNT signaling pathway [38]. We also computed the overlap of our human and mouse gene lists against the curated gene set in the MSigDB [39] gene set repository (Additional file 1: Tables S10-S13). This analysis identified signatures from multiple biological processes with relevance to cancer research including drug targets, chromosome maintenance, cellular proliferation, and therapeutic response.

## Discussion

When a mixed sample is analyzed using platforms specific to one species, it is possible that tissue from the other species will be detected, contaminating the assessment of which genes are present, or their levels of expression [40]. This is confirmed by our results demonstrating that although the overall levels of cross-hybridization and cross-alignment may be low, the specific genes that are affected are relevant to cancer research. Our cross-hybridization/cross-alignment lists contain a few thousand genes, some of which are known to play roles in biological processes related to cancer. Affected processes include EMT, Wnt signaling, DNA damage, and TGF-beta signaling.

The lists of affected genes and processes vary by species and platform. This is relevant to researchers in making decisions about how to process samples (LCM or no LCM?) and what tools to use to measure expression (microarray or NGS?). It is clear that such decisions are influenced not only by time and cost, but also by their impact on expression measurements. We have examined one context relevant to oncologic research, i.e., breast cancer pseudo-xenografts, and we surmise that examinations of other contexts will prove valuable to the research community.

## Conclusions

Oncology studies involving xenografts can be very useful in identifying genomic changes in both stroma and tumor that accompany the onset of disease and metastatic growth. Due to the small size of xenografts from metastatic tumors, and the expense of LCM, investigators who are interested in changes specific to stroma, or specific to tumor, may choose to select a gross sample (a mixture of stroma and tumor) and use a genomic platform that is specific to one species, either mouse or human.

The concern in this context is cross-hybridization (for microarrays) or cross-alignment (for RNA-Seq). Our results, using a definition of cross-hybridization/cross-alignment which should only identify genes with undetectable expression in same-species platforms but detectable expression in cross-species platforms - indicate that the overall percentage of genes affected by cross-hybridization and cross-alignment is small for both platforms. However, this percentage does include genes that are of interest to oncologic researchers, as they have known or purported roles in cancer progression and metastasis. The specific genes that cross-hybridize/cross-align are specific to the platform used to detect expression. As such the question of which platform to use with mixed tissues from xenografts – microarrays or high-throughput RNA sequencing – appears to be primarily a question of cost and the specific genes of research interest.

### Accession code

NCBI Gene Expression Omnibus: Microarray and RNA-Seq data has been deposited with accession number GSE40892.

## Competing interests

The authors declare that they have no competing interests.

## Authors’ contributions

CV carried out the bioinformatic analyses and helped draft the manuscript. PS helped design the study, carried out the biological experiments, and provided the expression data. NT helped design the study and guide the bioinformatic analyses. JC coordinated the study, guided the bioinformatic analyses, and participated in manuscript drafting. All authors read and approved the final manuscript.

## Supplementary Material

Additional file 1: Figure S1 – Detection Levels by Technology. **Figure S2 **– Detected CCDS IDs in 100% Samples. **Figure S3 **– Cross Alignment & Cross Hybridization. **Figure S4 **– RNA-Seq Alignments. **Figure S5 **– RNA-Seq CCDS Alignments. **Figure S6 **– Transcriptome Alignments. **Table S1** – Transcriptome Alignments. **Figure S7. **Cross-Aligning (RNA-Seq) Human GeneGo Pathway Maps using the CCDS ID gene catalog. **Figure S8. **Cross-Aligning (RNA-Seq) Mouse GeneGo Pathway Maps using the CCDS ID gene catalog. **Figure S9.** Cross-Hybridizing (Microarray) Human GeneGo Pathway Maps using the CCDS ID gene catalog. **Figure S10. **Cross-Hybridizing (Microarray) Mouse GeneGo Pathway Maps using the CCDS ID gene catalog. **Figure S11. **Cross-Hybridizing (Microarray) Human GeneGo Pathway Maps using a disjoint gene catalog. **Figure S12.** Cross-Hybridizing (Microarray) Mouse GeneGo Pathway Maps using a disjoint gene catalog. **Figure S13. **Cross-Aligning (RNA-Seq) Human GeneGo Pathway Maps using a disjoint gene catalog. **Figure S14. **Cross-Aligning (RNA-Seq) Mouse GeneGo Pathway Maps using a disjoint gene catalog. **Table S2 **– Human & Mouse CCDS Detection Levels by Technology. **Table S3 **– RNA-Seq Alignments. **Table S4 **– RNA-Seq CCDS Alignments. **Table S5 **– Detected CCDS IDs. **Table S6 **– Human & Mouse Cross Hybridizing Genes - Microarray. **Table S7 **– Human & Mouse Cross Aligning Genes – RNA-Seq. **Table S8 **– Human & Mouse Cross Hybridizing CCDS - Microarray. **Table S9 **– Human & Mouse Cross Aligning CCDS – RNA-Seq. **Table S10 **– Human Cross Alignment GSEA/MSigDB Analysis. **Table S11 **– Mouse Cross Alignment GSEA/MSigDB Analysis. **Table S12** – Human Cross Hybridization GSEA/MSigDB Analysis. **Table S13 **– Mouse Cross Hybridization GSEA/MSigDB Analysis. Click here for file

Additional file 2 List of Human cross-hybridizing and cross-aligning genes for microarray and RNA-Seq. Click here for file

Additional file 3 List of Mouse cross-hybridizing and cross-aligning genes found in microarrays and RNA-Seq. Click here for file
