# Supplementary material for: Characteristics of cross-hybridization and cross-alignment of expression in pseudo-xenograft samples by RNA-Seq and microarrays
Source: J Clin Bioinforma. 2013 Apr 18;3:8. doi: 10.1186/2043-9113-3-8 (PMC3667020; doi:10.1186/2043-9113-3-8)
Supplement: Additional file 1: Figure S1 — – Detection Levels by Technology. Figure S2 – Detected CCDS IDs in 100% Samples. Figure S3 – Cross Alignment & Cross Hybridization. Figure S4 – RNA-Seq Alignments. Figure S5 – RNA-Seq CCDS Alignments. Figure S6 – Transcriptome Alignments. Table S1 – Transcriptome Alignments. Figure S7. Cross-Aligning (RNA-Seq) Human GeneGo Pathway Maps using the CCDS ID gene catalog. Figure S8. Cross-Aligning (RNA-Seq) Mouse GeneGo Pathway Maps using the CCDS ID gene catalog. Figure S9. Cross-Hybridizing (Microarray) Human GeneGo Pathway Maps using the CCDS ID gene catalog. Figure S10. Cross-Hybridizing (Microarray) Mouse GeneGo Pathway Maps using the CCDS ID gene catalog. Figure S11. Cross-Hybridizing (Microarray) Human GeneGo Pathway Maps using a disjoint gene catalog. Figure S12. Cross-Hybridizing (Microarray) Mouse GeneGo Pathway Maps using a disjoint gene catalog. Figure S13. Cross-Aligning (RNA-Seq) Human GeneGo Pathway Maps using a disjoint gene catalog. Figure S14. Cross-Aligning (RNA-Seq) Mouse GeneGo Pathway Maps using a disjoint gene catalog. Table S2 – Human & Mouse CCDS Detection Levels by Technology. Table S3 – RNA-Seq Alignments. Table S4 – RNA-Seq CCDS Alignments. Table S5 – Detected CCDS IDs. Table S6 – Human & Mouse Cross Hybridizing Genes - Microarray. Table S7 – Human & Mouse Cross Aligning Genes – RNA-Seq. Table S8 – Human & Mouse Cross Hybridizing CCDS - Microarray. Table S9 – Human & Mouse Cross Aligning CCDS – RNA-Seq. Table S10 – Human Cross Alignment GSEA/MSigDB Analysis. Table S11 – Mouse Cross Alignment GSEA/MSigDB Analysis. Table S12 – Human Cross Hybridization GSEA/MSigDB Analysis. Table S13 – Mouse Cross Hybridization GSEA/MSigDB Analysis. [file 2043-9113-3-8-S1.doc]

**Supplemental**

**Characteristics of Cross-hybridization and Cross-alignment of Expression in Pseudo-Xenograft Samples by RNA-Seq and Microarrays**

**Camilo Valdes ^1^, Pearl Seo ^2^, Nicholas Tsinoremas ^1,4^, Jennifer Clarke ^3§^**

^1^Center for Computational Science, University of Miami, Miami, FL

^2^Department of Medicine, University of Miami, Miami, FL

^3^Division of Biostatistics, Department of Epidemiology and Public Health, University of Miami, Miami, FL

^4^Department of Computer Science, University of Miami, Miami, FL

*These authors contributed equally to this work

^§^Corresponding author

Email addresses:

CV: [CValdes3@med.miami.edu](mailto:cvaldes3@med.miami.edu)

PS: [PSeo@med.miami.edu](mailto:PSeo@med.miami.edu)

NT: [NTsinoremas@med.miami.edu](mailto:NTsinoremas@med.miami.edu)

JC: [JClarke@biostat.med.miami.edu](mailto:JClarke@biostat.med.miami.edu)

**Supplementary Figures & Tables**


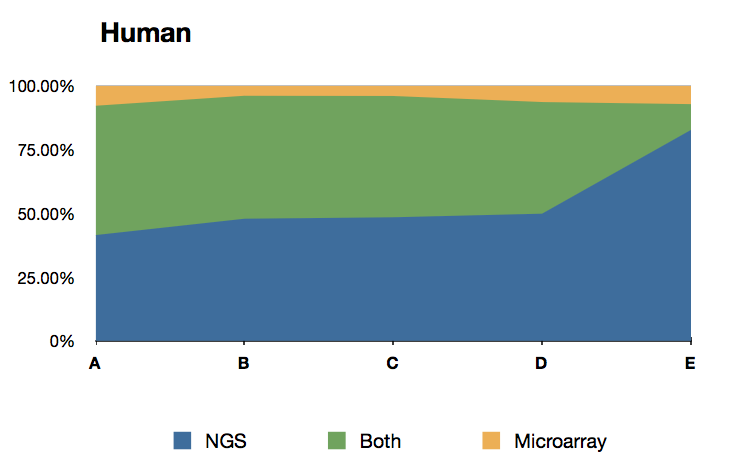

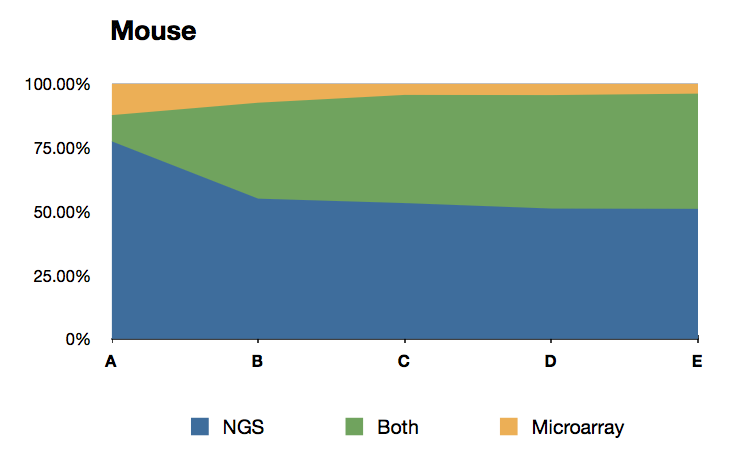


**Supplementary Figure 1 – Detection Levels by Technology**

Levels of CCDS IDs detected by RNA-Seq, microarrays, and both in each sample. The blue band represents CCDSs detected by RNA-Seq only; the green band represents a CCDS ID detected by both technologies; the yellow band represents a CCDS ID detected by microarrays only.


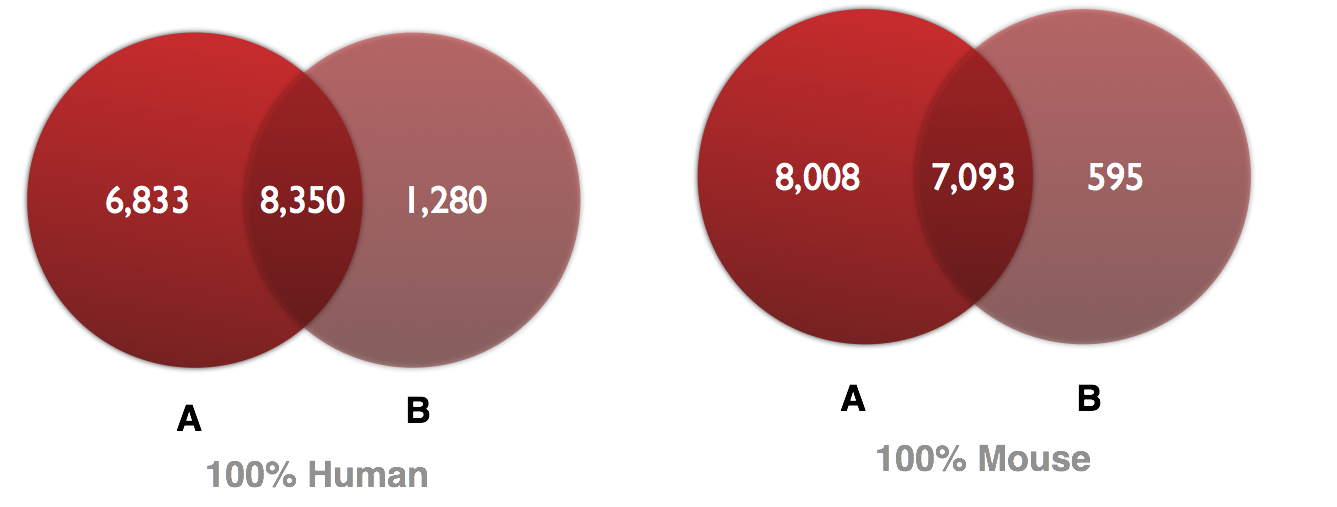


I

II

**Supplementary Figure 2 – Detected CCDS IDs in 100% Samples**

Homogeneous sample detection in 100% Human (A) and 100% Mouse (B) samples by aligning to the human genome and using the human chips (**I**). Homogeneous sample detection in 100% Human (A) and 100% Mouse (B) samples by aligning to the mouse genome and using the mouse chips (**II**).

I

II


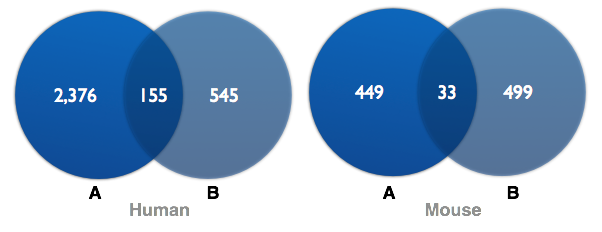


**Supplementary Figure 3 – Cross Alignment & Cross Hybridization**

Number of CCDS IDs that are identified as cross-aligning or cross-hybridizing and identified by RNA-Seq cross-alignments (A) and microarray cross-hybridizations (B) using human references (**I**). Number of CCDS IDs that are identified as cross-aligning or cross-hybridizing and identified by RNA-Seq cross-alignments (A) and microarray cross-hybridizations (B) using mouse references (**II**).


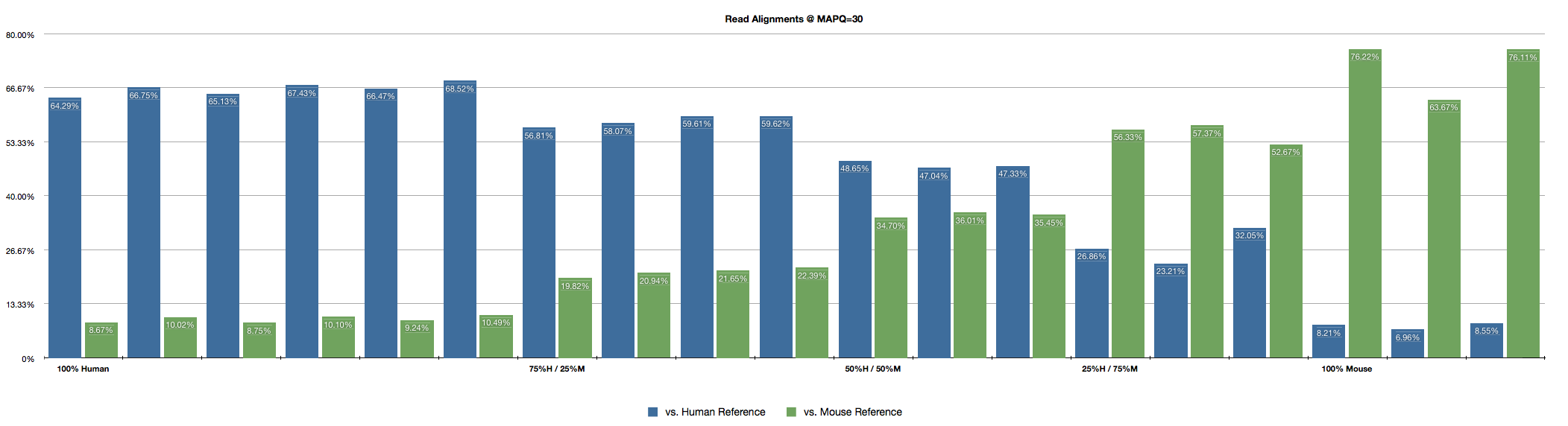


**Supplementary Figure 4 – RNA-Seq Alignments**

RNA-Seq alignments to human and mouse references. Alignments are filtered based on their mapping qualities (MAPQ=30).

**
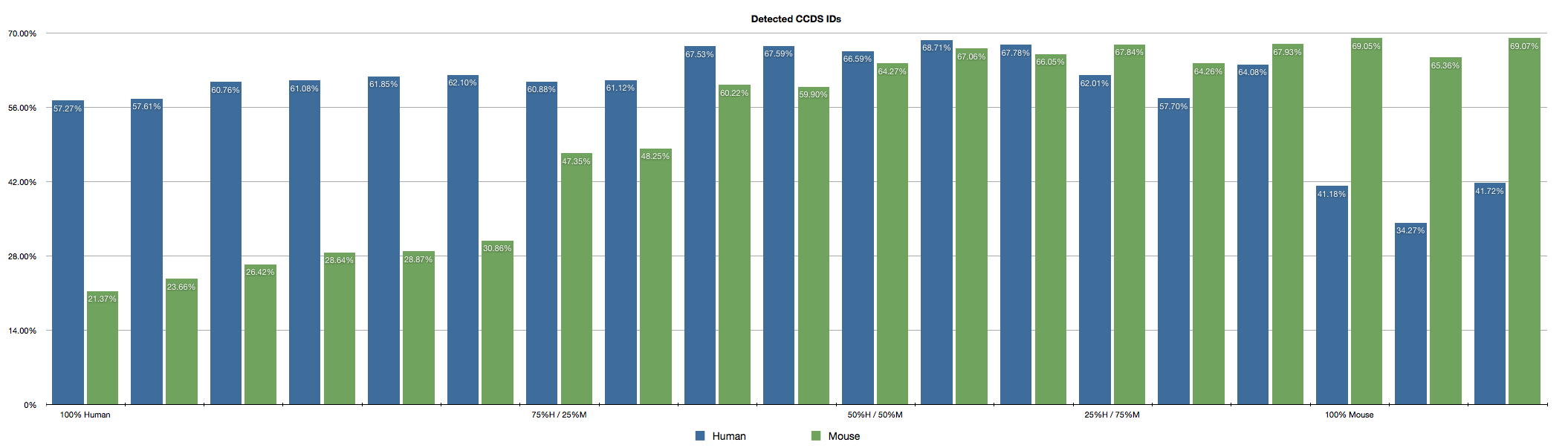
**

**Supplementary Figure 5 – RNA-Seq CCDS Alignments**

RNA-Seq alignments to human and mouse CCDS references. Alignments are filtered based on their mapping qualities (MAPQ=30).


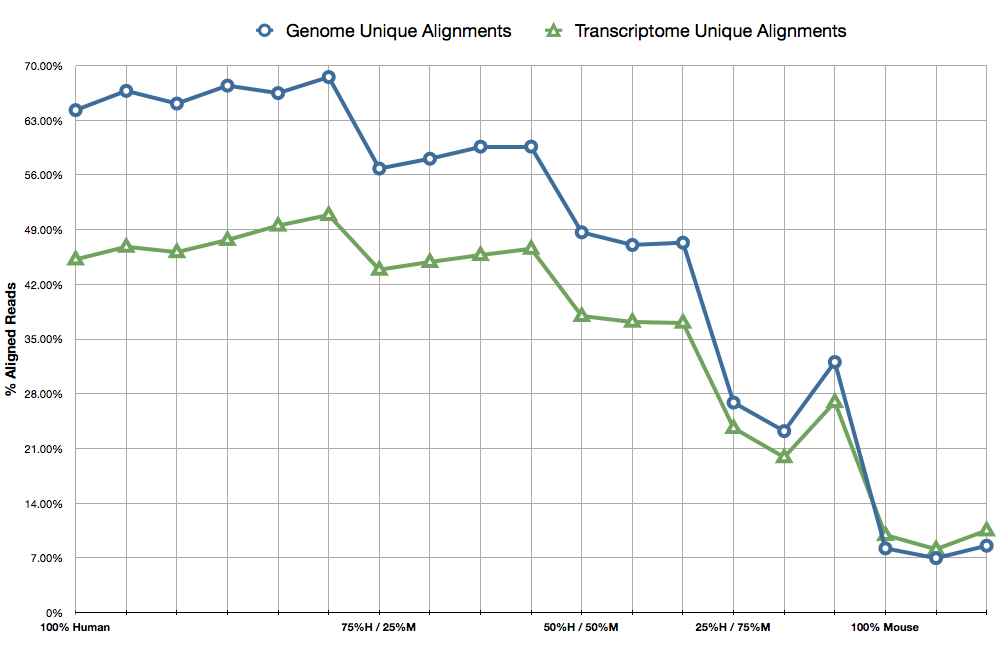


**Supplementary Figure 6 – Transcriptome Alignments**

Comparison of aligning samples to the human genome and transcriptome to gauge any advantages of aligning to either one.

**
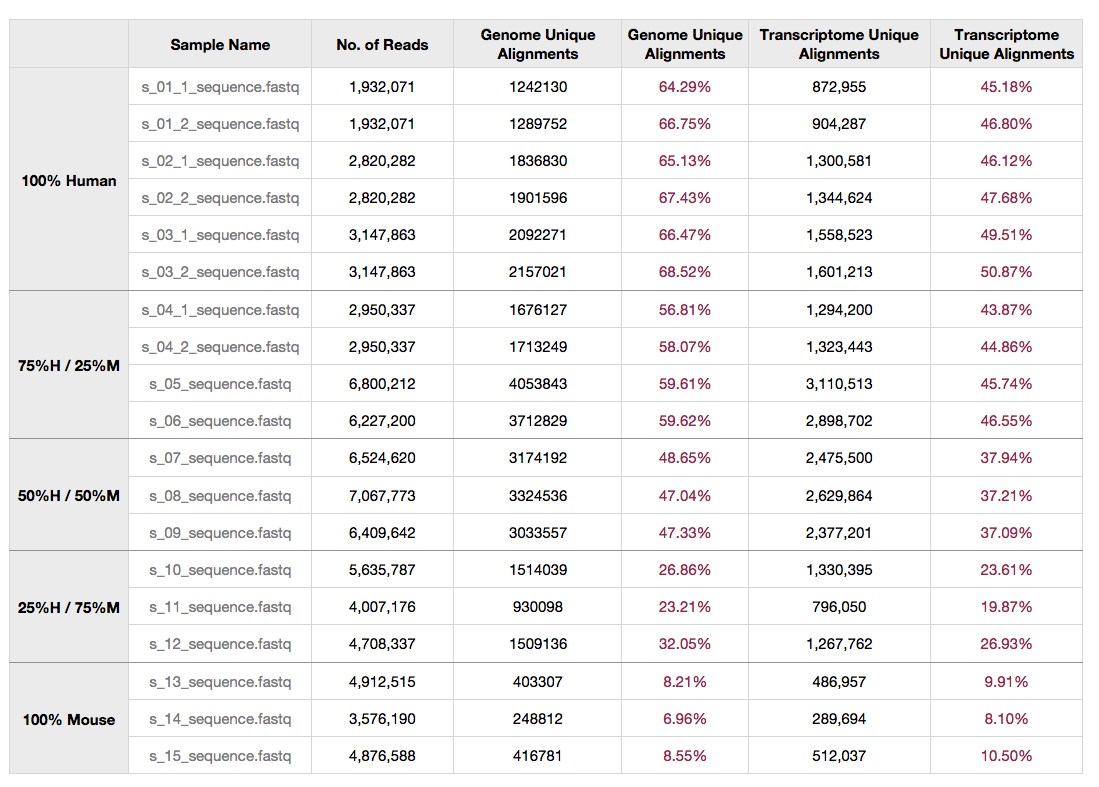
**

**Supplementary Table 1 – Transcriptome Alignments**

Results of aligning samples to the human genome and transcriptome to gauge any advantages of aligning to either one.

### GeneGo CCDS Analysis Pathway Maps

Canonical pathway maps represent a set of about 650 signaling and metabolic maps covering human biology (signaling and metabolism) in a comprehensive way. All maps are drawn from scratch by GeneGo annotators and manually curated & edited. Experimental data is visualized on the maps as blue (for downregulation) and red (upregulation) histograms. The height of the histogram corresponds to pathway map enrichment P-values for the genes analyzed (using –log10).


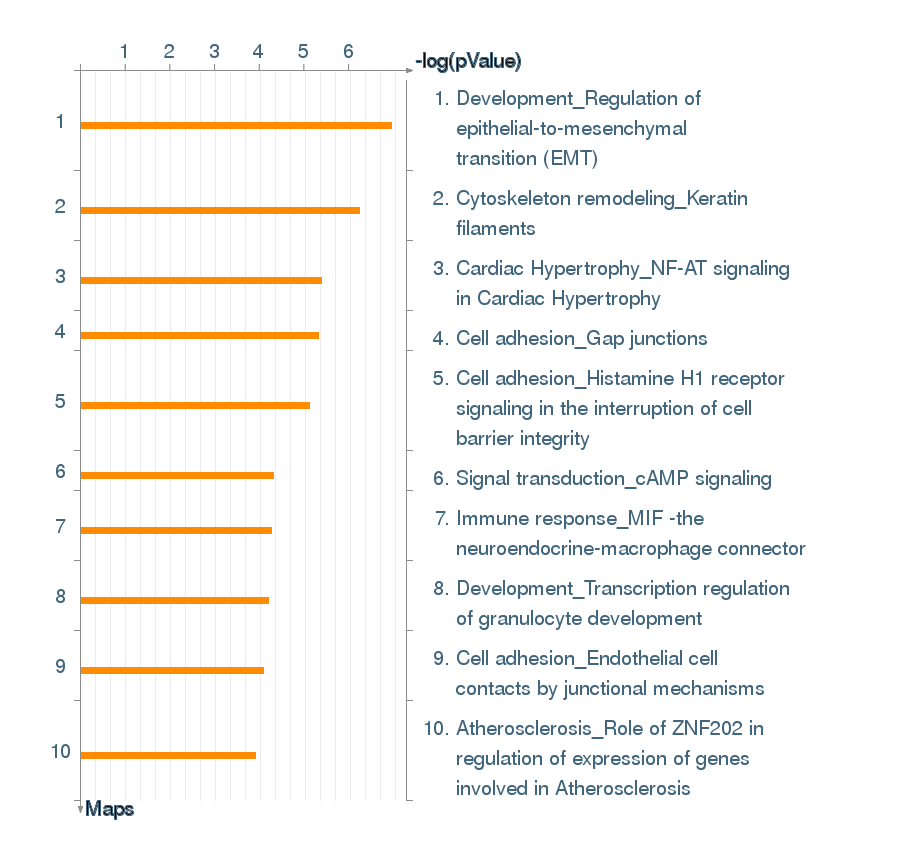


**Supplementary Figure 7.** **Cross-Aligning (RNA-Seq) Human GeneGo Pathway Maps using the CCDS ID gene catalog**.

Sorting is done for the 'Statistically significant Maps'.


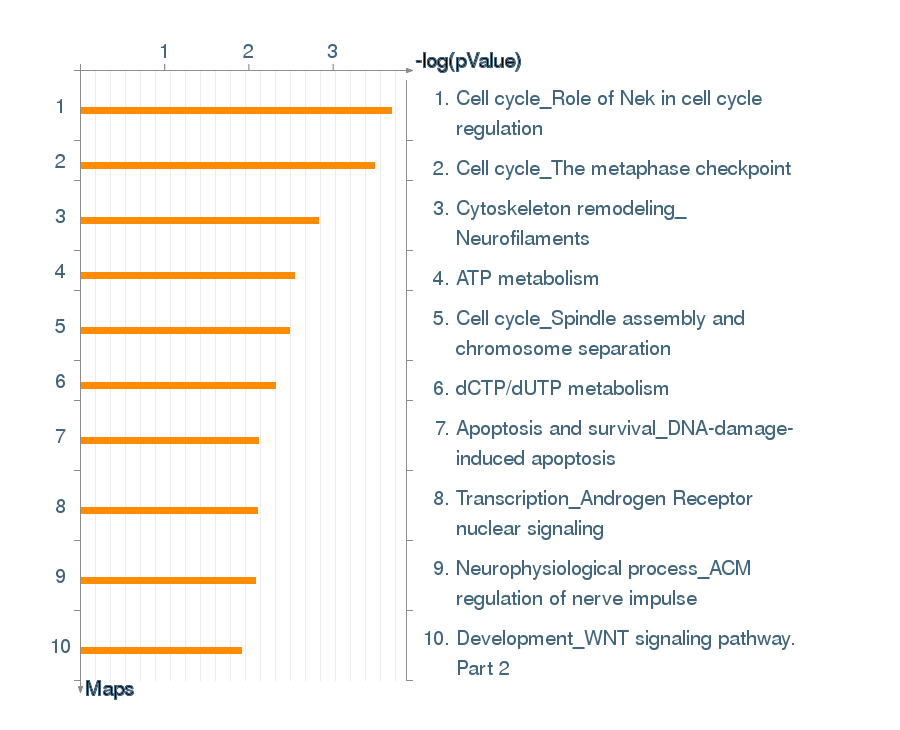


**Supplementary Figure 8.** **Cross-Aligning (RNA-Seq) Mouse GeneGo Pathway Maps using the CCDS ID gene catalog**.

Sorting is done for the 'Statistically significant Maps'.


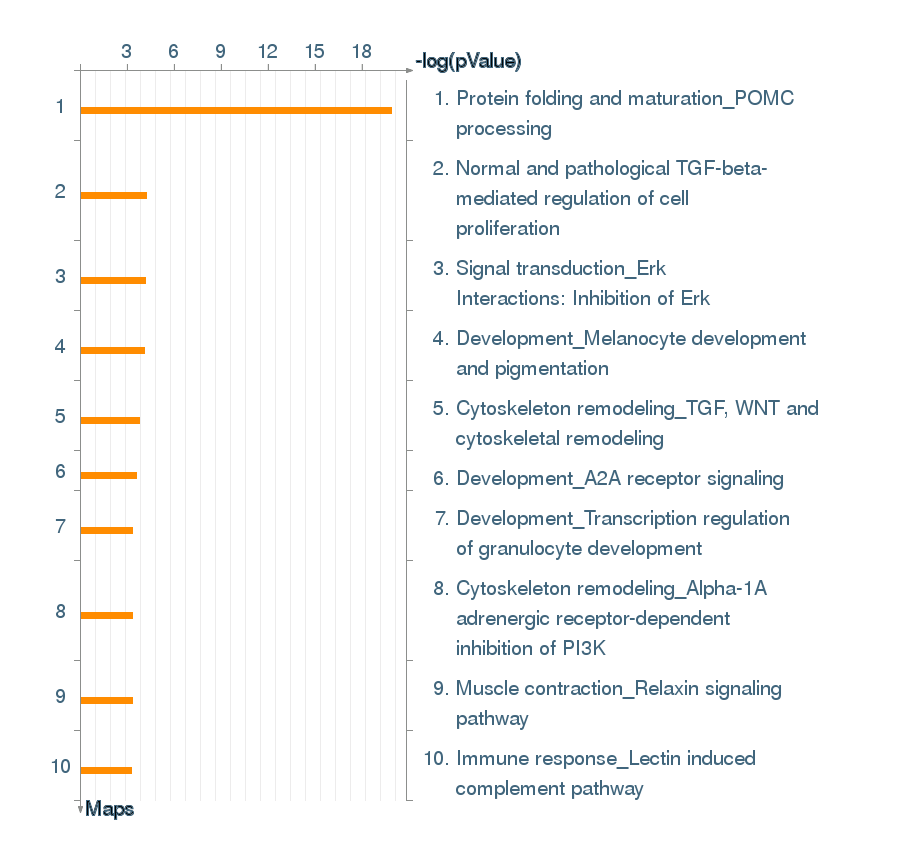


**Supplementary Figure 9.** **Cross-Hybridizing (Microarray) Human GeneGo Pathway Maps using the CCDS ID gene catalog**.

Sorting is done for the 'Statistically significant Maps'.


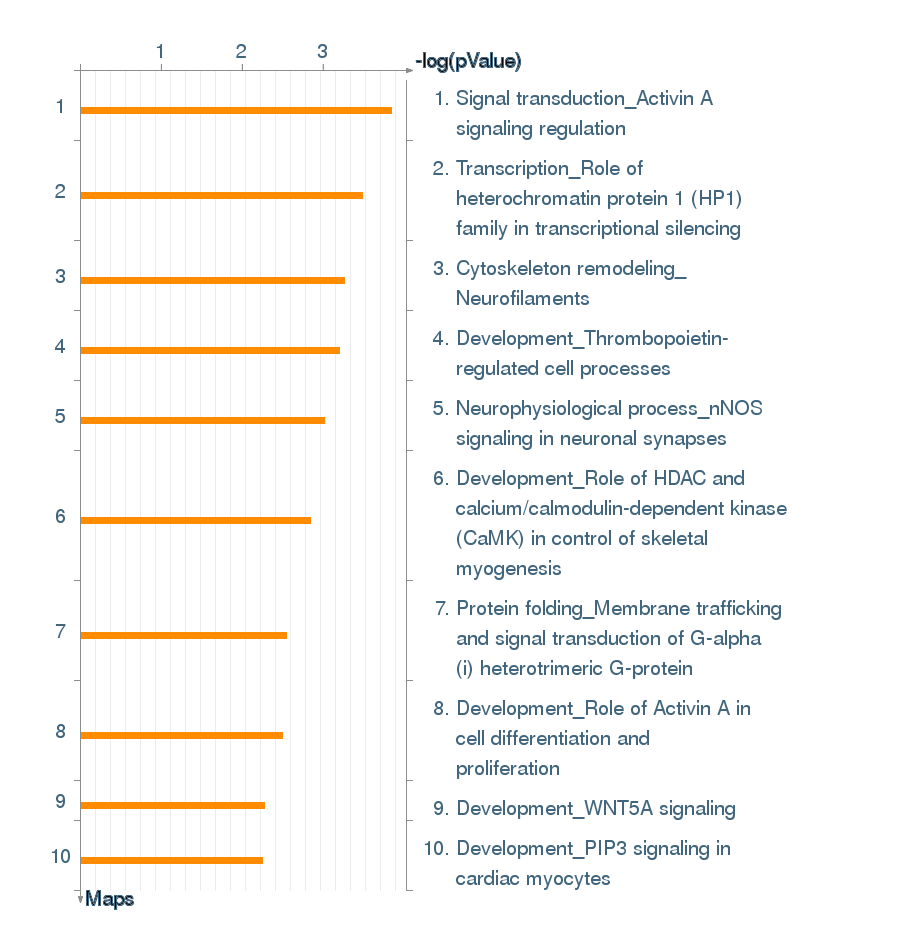


**Supplementary Figure 10.** **Cross-Hybridizing (Microarray) Mouse GeneGo Pathway Maps using the CCDS ID gene catalog**.

Sorting is done for the 'Statistically significant Maps'.

### GeneGo Disjoint-Gene Catalog Pathway Maps

Canonical pathway maps represent a set of about 650 signaling and metabolic maps covering human biology (signaling and metabolism) in a comprehensive way. All maps are drawn from scratch by GeneGo annotators and manually curated & edited. Experimental data is visualized on the maps as blue (for downregulation) and red (upregulation) histograms. The height of the histogram corresponds to pathway map enrichment P-values for the genes analyzed (using –log10).


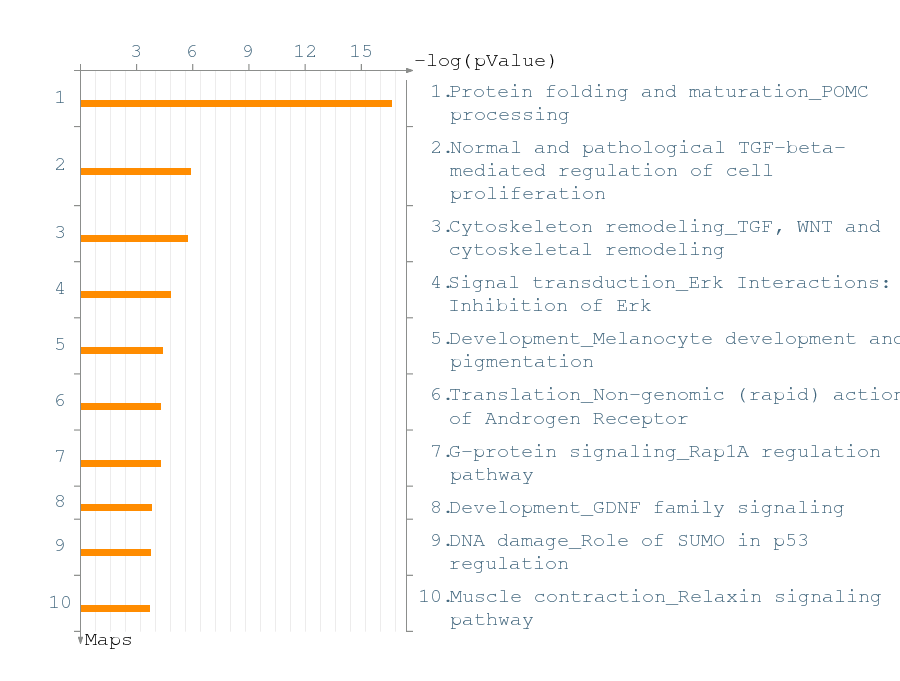


**Supplementary Figure 11.** **Cross-Hybridizing (Microarray) Human GeneGo Pathway Maps using a disjoint gene catalog**.

Sorting is done for the 'Statistically significant Maps'.


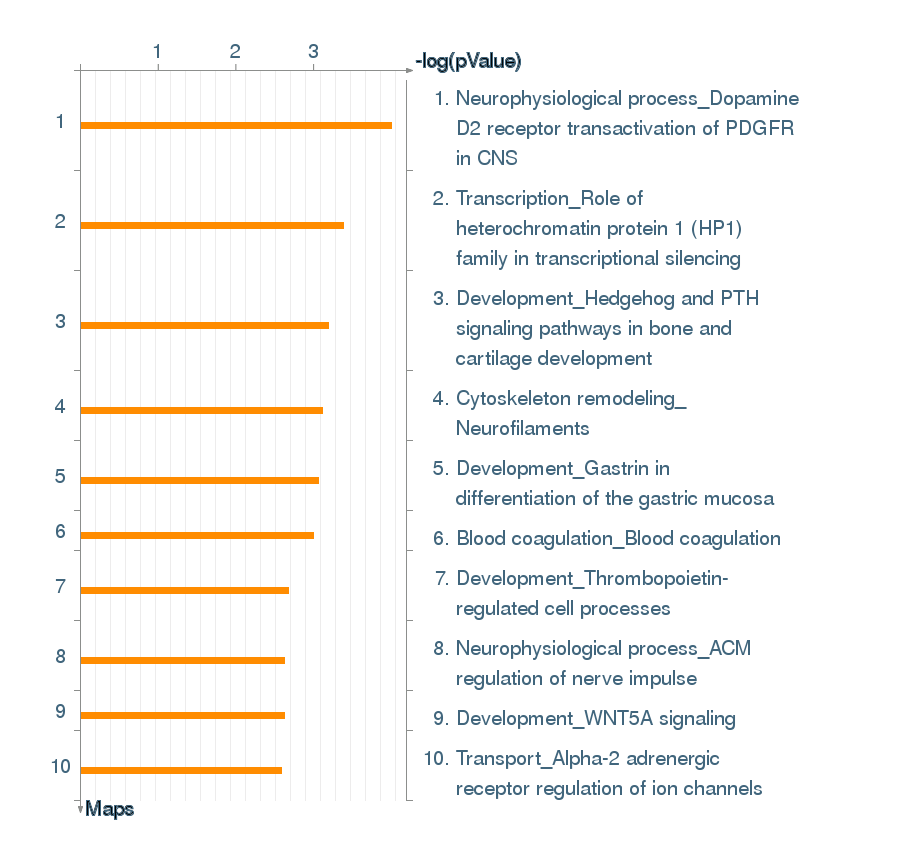


**Supplementary Figure 12.** **Cross-Hybridizing (Microarray) Mouse GeneGo Pathway Maps using a disjoint gene catalog**.

Sorting is done for the 'Statistically significant Maps'.


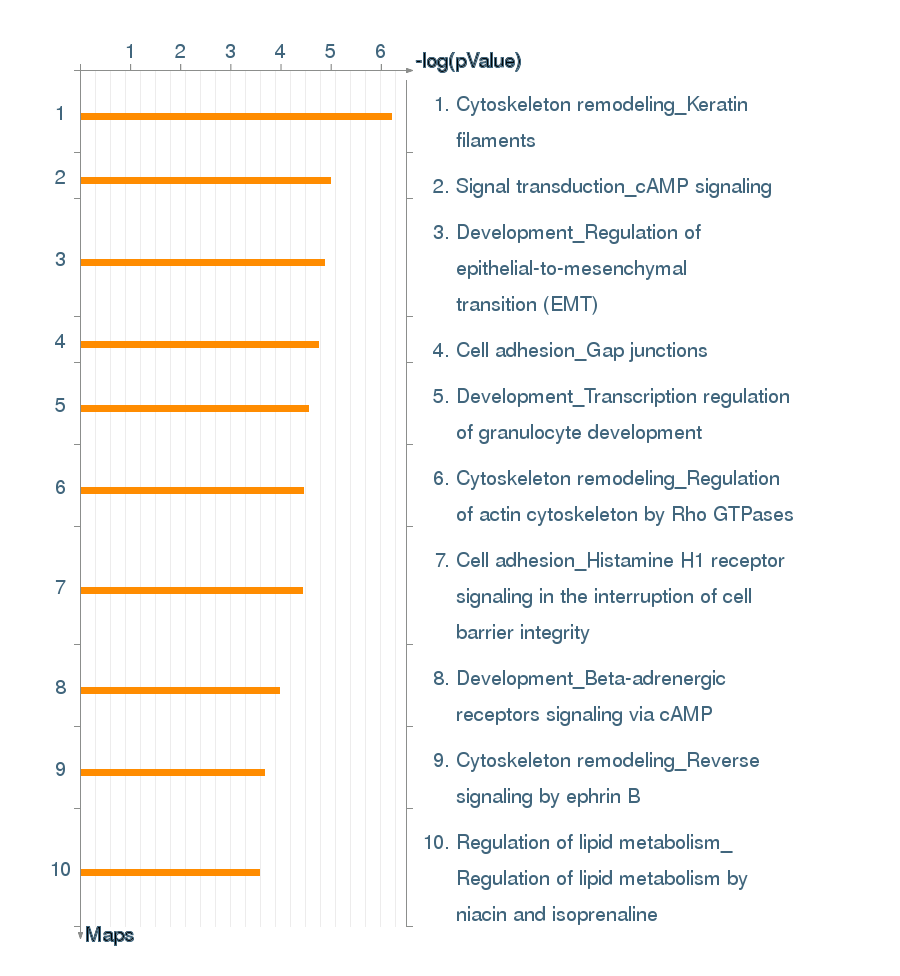


**Supplementary Figure 13.** **Cross-Aligning (RNA-Seq) Human GeneGo Pathway Maps using a disjoint gene catalog**.

Sorting is done for the 'Statistically significant Maps'.


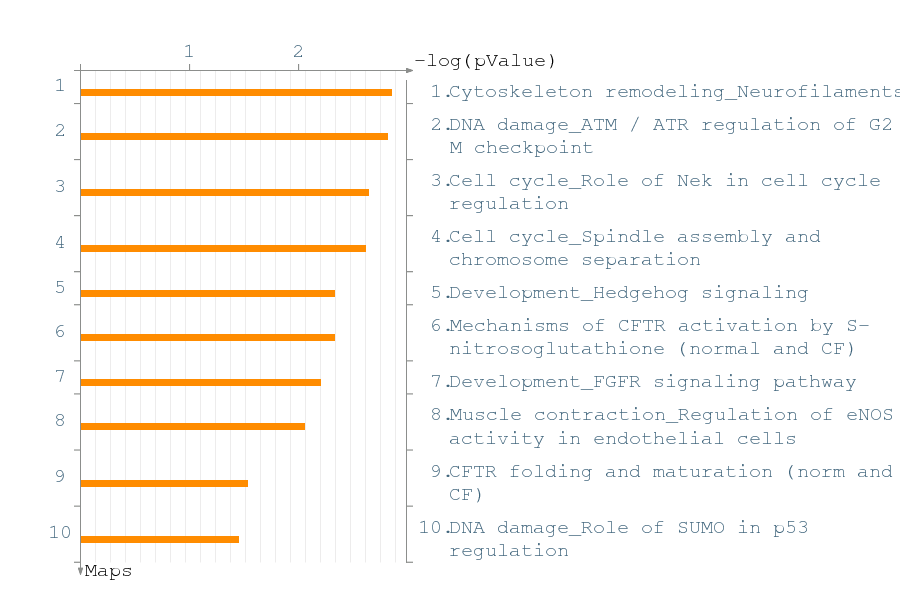


**Supplementary Figure 14.** **Cross-Aligning (RNA-Seq) Mouse GeneGo Pathway Maps using a disjoint gene catalog**.

Sorting is done for the 'Statistically significant Maps'

**
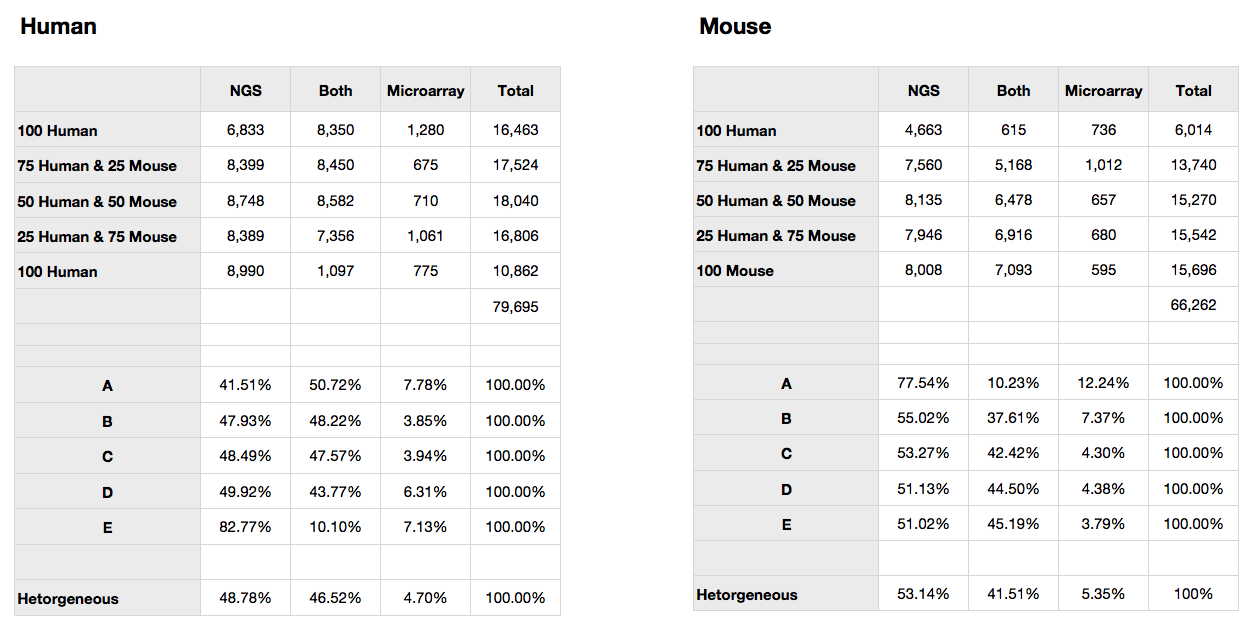
**

**Supplementary Table 2 – Human & Mouse CCDS Detection Levels by Technology**

Levels of CCDS IDs detected by RNA-Seq, microarrays, and both in each sample.


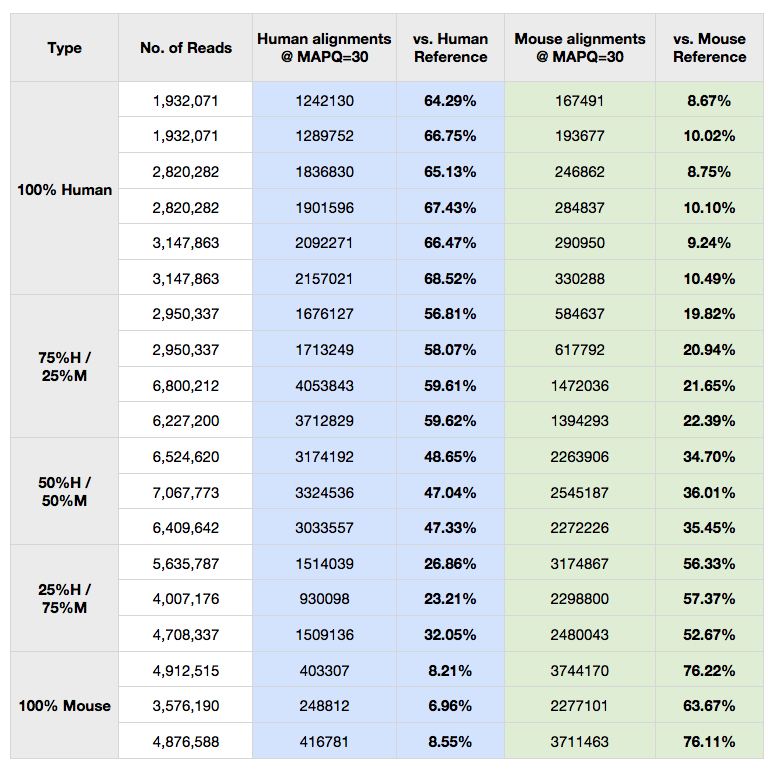


**Supplementary Table 3 – RNA-Seq Alignments**

RNA-Seq alignments to human and mouse references. Alignments are filtered based on their mapping qualities (MAPQ=30).


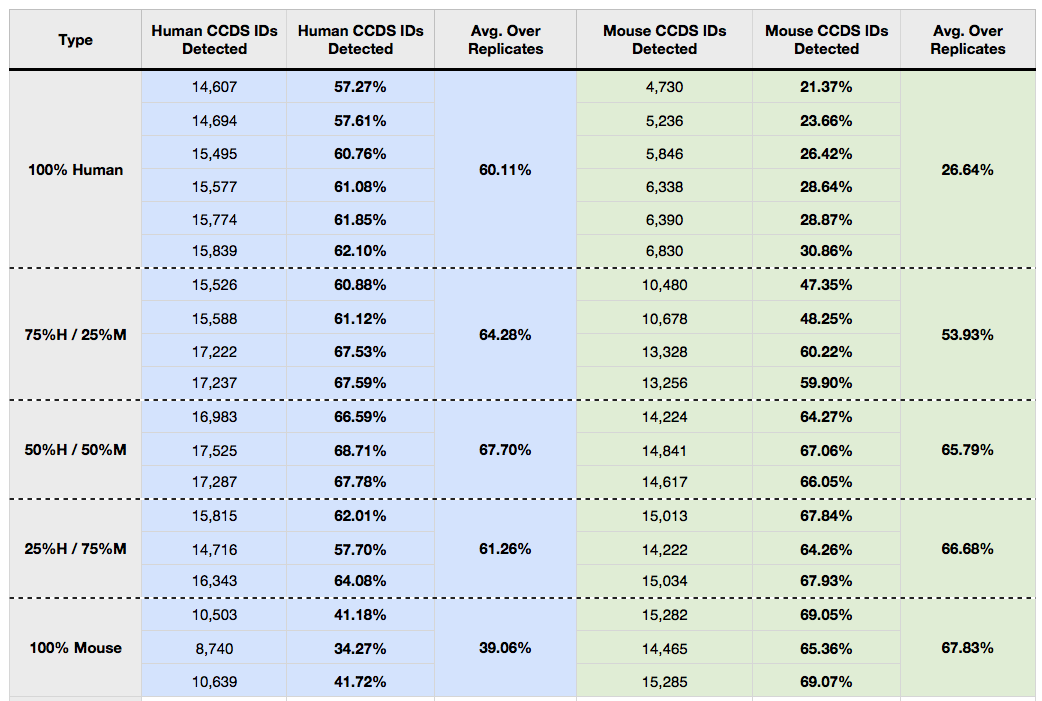


**Supplementary Table 4 – RNA-Seq CCDS Alignments**

RNA-Seq alignments to human and mouse CCDS references. Alignments are filtered based on their mapping qualities (MAPQ=30).


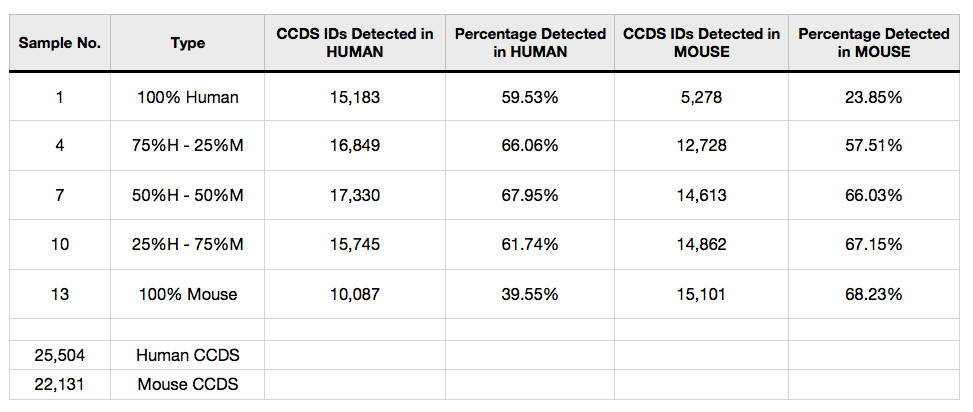


**Supplementary Table 5 – Detected CCDS IDs**

CCDS IDs detected in 2 out of 3 replicates.

|  | **Human** | **Mouse** |
| --- | --- | --- |
| **Sample E** | 4,162 | 2,536 |
| **Cross Hybridizers** | 2,597 | 1,574 |
| **Overlap** | 1,082 | 519 |
|  | **41.7%** | **33.0%** |

**Supplementary Table 6 – Human & Mouse Cross Hybridizing Genes - Microarray**

Cross hybridizing genes from the disjoint gene catalog. Sample E are those genes detected in the contrasting 100% sample, Cross Hybridizers are those genes detected using our method ((B ∪ C ∪ D) – A). Overlap are those genes common to both methods.

|  | **Human** | **Mouse** |
| --- | --- | --- |
| **Sample E** | 6,652 | 4,076 |
| **Cross Hybridizers** | 1,333 | 507 |
| **Overlap** | 604 | 88 |
|  | **45.3%** | **17.4%** |

**Supplementary Table 7 – Human & Mouse Cross Aligning Genes – RNA-Seq**

Cross aligning genes from the disjoint gene catalog. Sample E are those genes detected in the contrasting 100% sample, Cross Hybridizers are those genes detected using our method ((B ∪ C ∪ D) – A). Overlap are those genes common to both methods.

|  | **Human** | **Mouse** |
| --- | --- | --- |
| **Sample E** | 1,872 | 1,351 |
| **Cross Hybridizers** | 699 | 531 |
| **Overlap** | 248 | 128 |
|  | **35.5%** | **24.1%** |

**Supplementary Table 8 – Human & Mouse Cross Hybridizing CCDS - Microarray**

Cross hybridizing CCDS IDs from the CCDS catalog. Sample E are those CCDS IDs detected in the contrasting 100% sample, Cross Hybridizers are those CCDS IDs detected using our method ((B ∪ C ∪ D) – A). Overlap are those CCDS IDs common to both methods.

|  | **Human** | **Mouse** |
| --- | --- | --- |
| **Sample E** | 10,087 | 5,278 |
| **Cross Hybridizers** | 2,530 | 481 |
| **Overlap** | 1398 | 92 |
|  | **55.3%** | **19.1%** |

**Supplementary Table 9 – Human & Mouse Cross Aligning CCDS – RNA-Seq**

Cross aligning CCDS IDs from the CCDS catalog. Sample E are those CCDS IDs detected in the contrasting 100% sample, Cross Hybridizers are those CCDS IDs detected using our method ((B ∪ C ∪ D) – A). Overlap are those CCDS IDs common to both methods.

| **Gene Set Name** | **# Genes in Gene Set (K)** | **# Genes in Overlap (k)** | **k/K** | **p value** |
| --- | --- | --- | --- | --- |
| BENPORATH_EED_TARGETS | 1062 | 170 | 0.1601 | 0.00E+00 |
| MEISSNER_BRAIN_HCP_WITH_H3K4ME3_AND_H3K27ME3 | 1069 | 186 | 0.1721 | 0.00E+00 |
| BENPORATH_SUZ12_TARGETS | 1038 | 186 | 0.1792 | 0.00E+00 |
| BENPORATH_ES_WITH_H3K27ME3 | 1118 | 203 | 0.1807 | 0.00E+00 |
| SMID_BREAST_CANCER_NORMAL_LIKE_UP | 476 | 105 | 0.2185 | 0.00E+00 |
| LIM_MAMMARY_STEM_CELL_UP | 489 | 111 | 0.2209 | 0.00E+00 |
| ACEVEDO_FGFR1_TARGETS_IN_PROSTATE_CANCER_MODEL_DN | 308 | 76 | 0.2403 | 0.00E+00 |
| DELYS_THYROID_CANCER_DN | 232 | 65 | 0.2759 | 0.00E+00 |
| BOQUEST_STEM_CELL_UP | 260 | 76 | 0.2923 | 0.00E+00 |
| SWEET_LUNG_CANCER_KRAS_DN | 435 | 136 | 0.3103 | 0.00E+00 |
| BENPORATH_PRC2_TARGETS | 652 | 118 | 0.181 | 2.22E-16 |
| LEE_BMP2_TARGETS_UP | 745 | 132 | 0.1732 | 3.33E-16 |
| RICKMAN_HEAD_AND_NECK_CANCER_F | 54 | 27 | 0.5 | 9.99E-16 |
| BOQUEST_STEM_CELL_CULTURED_VS_FRESH_UP | 425 | 89 | 0.2047 | 1.44E-15 |
| VART_KSHV_INFECTION_ANGIOGENIC_MARKERS_UP | 165 | 47 | 0.2848 | 1.89E-14 |
| SCHUETZ_BREAST_CANCER_DUCTAL_INVASIVE_UP | 351 | 76 | 0.2108 | 3.90E-14 |
| WEST_ADRENOCORTICAL_TUMOR_DN | 546 | 101 | 0.1813 | 5.43E-14 |
| RIGGI_EWING_SARCOMA_PROGENITOR_UP | 430 | 85 | 0.1953 | 6.89E-14 |
| LINDGREN_BLADDER_CANCER_CLUSTER_2B | 392 | 79 | 0.2015 | 6.92E-14 |
| KUNINGER_IGF1_VS_PDGFB_TARGETS_UP | 82 | 31 | 0.378 | 1.17E-13 |

**Supplementary Table 10 – Human Cross Alignment GSEA/MSigDB Analysis**

Computed overlap of human cross aligners against the GSEA/MSigDB “curated gene sets”: Chemical and Genetic Perturbations, Canonical Pathways, KEGG gene sets, and REACTOME gene sets.

| **Gene Set Name** | **# Genes in Gene Set (K)** | **# Genes in Overlap (k)** | **k/K** | **p value** |
| --- | --- | --- | --- | --- |
| BENPORATH_EED_TARGETS | 1062 | 46 | 0.0433 | 7.57E-11 |
| BENPORATH_ES_WITH_H3K27ME3 | 1118 | 47 | 0.042 | 1.25E-10 |
| BENPORATH_SUZ12_TARGETS | 1038 | 44 | 0.0424 | 4.07E-10 |
| MIKKELSEN_MCV6_HCP_WITH_H3K27ME3 | 435 | 27 | 0.0621 | 4.81E-10 |
| MEISSNER_BRAIN_HCP_WITH_H3K4ME3_AND_H3K27ME3 | 1069 | 43 | 0.0402 | 3.20E-09 |
| KOBAYASHI_EGFR_SIGNALING_24HR_DN | 251 | 18 | 0.0717 | 4.71E-08 |
| BENPORATH_PRC2_TARGETS | 652 | 30 | 0.046 | 5.26E-08 |
| DUTERTRE_ESTRADIOL_RESPONSE_24HR_UP | 324 | 20 | 0.0617 | 1.01E-07 |
| ROSTY_CERVICAL_CANCER_PROLIFERATION_CLUSTER | 140 | 13 | 0.0929 | 1.86E-07 |
| MEISSNER_NPC_HCP_WITH_H3K4ME2_AND_H3K27ME3 | 349 | 20 | 0.0573 | 3.32E-07 |
| HAN_SATB1_TARGETS_UP | 395 | 21 | 0.0532 | 5.77E-07 |
| VECCHI_GASTRIC_CANCER_EARLY_UP | 430 | 22 | 0.0512 | 5.91E-07 |
| MEISSNER_NPC_HCP_WITH_H3K4ME3_AND_H3K27ME3 | 142 | 12 | 0.0845 | 1.52E-06 |
| GOBERT_OLIGODENDROCYTE_DIFFERENTIATION_UP | 570 | 25 | 0.0439 | 1.70E-06 |
| FUJII_YBX1_TARGETS_DN | 202 | 14 | 0.0693 | 2.23E-06 |
| MIKKELSEN_NPC_HCP_WITH_H3K27ME3 | 341 | 18 | 0.0528 | 4.14E-06 |
| HORIUCHI_WTAP_TARGETS_DN | 310 | 16 | 0.0516 | 1.87E-05 |
| MIKKELSEN_MEF_HCP_WITH_H3K27ME3 | 590 | 23 | 0.039 | 2.94E-05 |
| RODRIGUES_THYROID_CARCINOMA_ANAPLASTIC_UP | 722 | 26 | 0.036 | 3.43E-05 |
| FERREIRA_EWINGS_SARCOMA_UNSTABLE_VS_STABLE_UP | 167 | 11 | 0.0659 | 4.38E-05 |

**Supplementary Table 11 – Mouse Cross Alignment GSEA/MSigDB Analysis**

Computed overlap of mouse cross aligners against the GSEA/MSigDB “curated gene sets”: Chemical and Genetic Perturbations, Canonical Pathways, KEGG gene sets, and REACTOME gene sets

| **Gene Set Name** | **# Genes in Gene Set (K)** | **# Genes in Overlap (k)** | **k/K** | **p value** |
| --- | --- | --- | --- | --- |
| BENPORATH_ES_WITH_H3K27ME3 | 1118 | 67 | 0.0599 | 1.06E-07 |
| BENPORATH_SUZ12_TARGETS | 1038 | 59 | 0.0568 | 3.46E-06 |
| IVANOVA_HEMATOPOIESIS_STEM_CELL_AND_PROGENITOR | 681 | 45 | 0.0631 | 6.26E-06 |
| REACTOME_AMYLOIDS | 83 | 12 | 0.1446 | 7.97E-06 |
| REACTOME_MEIOSIS | 116 | 14 | 0.1207 | 1.23E-05 |
| REACTOME_MEIOTIC_SYNAPSIS | 73 | 11 | 0.1507 | 1.27E-05 |
| MEISSNER_NPC_HCP_WITH_H3K4ME2 | 491 | 32 | 0.0652 | 5.29E-05 |
| LEE_LIVER_CANCER_DENA_DN | 74 | 10 | 0.1351 | 8.16E-05 |
| KEGG_SYSTEMIC_LUPUS_ERYTHEMATOSUS | 140 | 14 | 0.1 | 1.01E-04 |
| REACTOME_RNA_POL_I_PROMOTER_OPENING | 62 | 9 | 0.1452 | 1.05E-04 |
| MIKKELSEN_MEF_HCP_WITH_H3K27ME3 | 590 | 35 | 0.0593 | 1.56E-04 |
| MARTENS_TRETINOIN_RESPONSE_UP | 857 | 48 | 0.0537 | 1.62E-04 |
| BENPORATH_EED_TARGETS | 1062 | 55 | 0.0508 | 1.77E-04 |
| DELYS_THYROID_CANCER_DN | 232 | 18 | 0.0776 | 2.95E-04 |
| GEORGANTAS_HSC_MARKERS | 71 | 9 | 0.1268 | 3.02E-04 |
| SMID_BREAST_CANCER_LUMINAL_B_DN | 564 | 33 | 0.0585 | 3.05E-04 |
| MIKKELSEN_NPC_HCP_WITH_H3K27ME3 | 341 | 23 | 0.0674 | 3.58E-04 |
| REACTOME_CHROMOSOME_MAINTENANCE | 122 | 12 | 0.0984 | 3.63E-04 |
| WANG_SMARCE1_TARGETS_UP | 280 | 20 | 0.0714 | 4.12E-04 |
| BALLIF_DEVELOPMENTAL_DISABILITY_P16_P12_DELETION | 13 | 4 | 0.3077 | 4.95E-04 |

**Supplementary Table 12 – Human Cross Hybridization GSEA/MSigDB Analysis**

Computed overlap of human cross hybridizers against the GSEA/MSigDB “curated gene sets”: Chemical and Genetic Perturbations, Canonical Pathways, KEGG gene sets, and REACTOME gene sets

| **Gene Set Name** | **# Genes in Gene Set (K)** | **# Genes in Overlap (k)** | **k/K** | **p value** |
| --- | --- | --- | --- | --- |
| BENPORATH_ES_WITH_H3K27ME3 | 1118 | 42 | 0.0376 | 4.30E-06 |
| OSADA_ASCL1_TARGETS_UP | 46 | 7 | 0.1522 | 1.59E-05 |
| KAYO_AGING_MUSCLE_UP | 244 | 15 | 0.0615 | 3.48E-05 |
| YOSHIMURA_MAPK8_TARGETS_UP | 1305 | 44 | 0.0337 | 3.64E-05 |
| REACTOME_GPCR_LIGAND_BINDING | 408 | 20 | 0.049 | 4.87E-05 |
| MEISSNER_NPC_HCP_WITH_H3K4ME2_AND_H3K27ME3 | 349 | 18 | 0.0516 | 6.06E-05 |
| MIKKELSEN_MEF_HCP_WITH_H3K27ME3 | 590 | 25 | 0.0424 | 6.35E-05 |
| DUAN_PRDM5_TARGETS | 79 | 8 | 0.1013 | 8.17E-05 |
| MIKKELSEN_IPS_HCP_WITH_H3_UNMETHYLATED | 80 | 8 | 0.1 | 8.94E-05 |
| BENPORATH_SUZ12_TARGETS | 1038 | 36 | 0.0347 | 1.11E-04 |
| REACTOME_NEURONAL_SYSTEM | 279 | 15 | 0.0538 | 1.57E-04 |
| HOSHIDA_LIVER_CANCER_SURVIVAL_DN | 113 | 9 | 0.0796 | 1.93E-04 |
| GRESHOCK_CANCER_COPY_NUMBER_UP | 323 | 16 | 0.0495 | 2.44E-04 |
| PID_AP1_PATHWAY | 70 | 7 | 0.1 | 2.46E-04 |
| REACTOME_POTASSIUM_CHANNELS | 98 | 8 | 0.0816 | 3.68E-04 |
| KEGG_NEUROACTIVE_LIGAND_RECEPTOR_INTERACTION | 272 | 14 | 0.0515 | 4.01E-04 |
| KIM_WT1_TARGETS_UP | 214 | 12 | 0.0561 | 4.83E-04 |
| SMID_BREAST_CANCER_BASAL_UP | 648 | 24 | 0.037 | 6.38E-04 |
| HERNANDEZ_ABERRANT_MITOSIS_BY_DOCETACEL_4NM_UP | 23 | 4 | 0.1739 | 6.72E-04 |
| BRUECKNER_TARGETS_OF_MIRLET7A3_UP | 111 | 8 | 0.0721 | 8.45E-04 |

**Supplementary Table 13 – Mouse Cross Hybridization GSEA/MSigDB Analysis**

Computed overlap of mouse cross hybridizers against the GSEA/MSigDB “curated gene sets”: Chemical and Genetic Perturbations, Canonical Pathways, KEGG gene sets, and REACTOME gene sets
